# Synovial Sarcoma: Molecular Biology, Pathology, and Therapeutic Strategies

**DOI:** 10.3390/ijms27094125

**Published:** 2026-05-05

**Authors:** Joon Hyuk Choi

**Affiliations:** Department of Pathology, College of Medicine, Yeungnam University, Daegu 42415, Republic of Korea; joonhyukchoi@ynu.ac.kr; Tel.: +82-53-640-6754

**Keywords:** synovial sarcoma, molecular pathology, tumor classification, diagnosis, treatment

## Abstract

Synovial sarcoma (SS) is a malignant mesenchymal neoplasm with variable epithelial differentiation. SS is defined by the presence of a specific *SS18*::*SSX* fusion gene. Moreover, SS can occur at any age, shows no significant sex predilection, and most commonly arises in the deep soft tissues of the extremities, typically in juxta-articular locations (approximately 70% of cases), with a variable prognosis. SS accounts for 5–10% of all soft tissue sarcomas. Meanwhile, despite recent advances in diagnosis and therapy, SS remains a diagnostic and clinical challenge due to the associated broad anatomical distribution and diverse histological patterns. This review aims to provide a comprehensive update on the clinical, molecular, and pathological features of SS, with emphasis on diagnostic strategies and treatment approaches.

## 1. Introduction

Synovial sarcoma (SS) is a malignant mesenchymal neoplasm characterized by variable epithelial differentiation and a defining *SS18*::*SSX1/2/4* fusion gene [1]. SS accounts for approximately 5–10% of all soft tissue sarcomas. Although SS can occur at any age and shows no sex predilection, this cancer predominantly affects adolescents and young adults, with approximately 77% of cases diagnosed before the age of 50. Most tumors (approximately 70%) arise in the deep soft tissues of the upper and lower extremities, frequently in juxta-articular locations [2]; however, SS can occur at virtually any anatomical site [3]. SS exhibits variable clinical behavior.

Although early literature suggested a microscopic resemblance between SS and synovium, there is no convincing evidence that SS originates from or differentiates toward synovial tissue [4,5,6]. Several archaic synonyms have been used historically, including tendosynovial sarcoma, synovial cell sarcoma, carcinosarcoma of soft tissues, malignant synovioma, and synovioblastic sarcoma [7,8,9]. However, use of these alternative terms is no longer recommended. SS exhibits a broad histological spectrum and has traditionally been classified into monophasic, biphasic, and poorly differentiated subtypes.

Advances in molecular genetics have improved understanding of the biology, classification, prognosis, and therapeutic strategies of SS. Nevertheless, SS continues to pose significant diagnostic and therapeutic challenges due to the associated histological and molecular heterogeneity, particularly in small biopsies and at unusual anatomic sites. Therefore, accurate pathological classification and molecular characterization are essential for optimal patient management and prognostic assessment in SS.

This review provides a comprehensive update on the pathology of SS, with emphasis on molecular genetics, histological features, and key differential diagnoses.

## 2. Historical Aspects

The earliest account attributed to SS is often credited to Simon, who in 1865 described a pedunculated tumor of the knee [10]. However, the illustrations in the report are now considered more consistent with a giant cell tumor or villonodular synovitis. The first convincing morphological description of typical SS is therefore generally attributed to Lejars and Rubens-Duval in 1910 [11], who documented a knee tumor with a biphasic epithelial and spindle cell pattern and called it “synovial endothelioma.” However, Smith [12] later questioned this designation and emphasized that such tumors arose not only in joints and bursae but also in tendon sheaths, fasciae, and aponeuroses, preferring the term “synovioma” because this terminology was descriptively useful yet histogenetically noncommittal.

The term SS was formally introduced in French in 1934 [13] and subsequently in English by Knox [14], who distinguished SS from fibrosarcoma based on the presence of an epithelial component. Thereafter, SS was defined by the associated characteristic biphasic morphology, while the subsequent clinical features were established through multiple case series [15,16,17]. The subsequent recognition of extra-articular cases arising in sites lacking synovial tissue raised increasing doubt about a true synovial origin. Consequently, SS came to be regarded as a mesenchymal neoplasm with epithelial differentiation rather than a tumor derived from the synovium [18]. A substantial number of spindle cell sarcomas previously classified as fibrosarcomas are now believed to represent monophasic SS, as first recognized in the early 1980s [19,20].

Although the existence of monophasic SS composed entirely of spindle cells was initially controversial [21], subsequent ultrastructural and immunohistochemical studies demonstrated epithelial features within these tumors [22], and shared cytogenetic and molecular genetic alterations in both monophasic and biphasic forms ultimately confirmed their biological unity.

## 3. Epidemiology

SS can occur at any age and shows no significant sex predilection. More than half of patients are adolescents or young adults, and approximately 77% of cases are diagnosed before the age of 50. Therefore, the relative frequency of SS is strongly age-dependent, accounting for approximately 15% of soft tissue sarcomas in patients aged 10–18 years but only 1.6% in those older than 50 years [2]. Accordingly, SS is the most common non-rhabdomyosarcoma (non-RMS) soft tissue sarcoma in children and young adults [23]. Most tumors (approximately 70%) arise in the deep soft tissue of the lower or upper extremities, typically in juxta-articular locations, whereas the trunk and head and neck region account for approximately 15% and 7% of cases, respectively. SS has also been reported at a wide range of unusual anatomic sites, including the retroperitoneum, gastrointestinal (GI) tract, lung, heart, mediastinum, urinary tract, male genital tract, bone, skin, female genital tract, central nervous system (predominantly extra-axial, with rare intra-axial involvement), and peripheral nerves [24,25,26,27,28,29,30,31].

Primary GI SS is exceedingly rare and most frequently involves the stomach, with only occasional cases reported in the esophagus, small intestine, and colon [32]. Moreover, primary GI SS predominantly affects middle-aged or older adults and similarly shows no sex predilection, although pediatric cases have been described, particularly in the esophagus. Primary intrathoracic SS is also uncommon, with more than 80% of cases arising in the pleura or pulmonary parenchyma; primary involvement of the mediastinum or heart is distinctly rare [33,34,35]. In comparison, primary intrathoracic SS shows a slight male predilection and occurs across a wide age range, with peak incidence in the fourth to fifth decades of life [36]. SS of the genitourinary tract is exceptionally rare, affects patients across a broad age range (15–78 years), and peaks during the third to fourth decades of life [37,38,39,40,41]. Most reported genitourinary cases arise in the kidney and prostate.

SS of the head and neck region is more common in young adults than in children and shows a male predominance [42,43,44]. Common sites include the parapharyngeal space, oropharynx, hypopharynx, and larynx. In contrast, ocular adnexal SS is rare and shows a female predominance, with a mean age of approximately 25 years [45]. Primary ocular adnexal SS has been reported in the epibulbar region, anterior orbit, and intraconal space, often with adherence to the extraocular muscles but without significant bone destruction [46]. Rarely, the orbit may be secondarily involved by metastatic SS [47].

## 4. Clinical Features

The clinical presentation of SS is often nonspecific and largely dependent on the anatomical site. Most patients present with a slowly enlarging mass that may be longstanding and occasionally painful. Early tumor growth is frequently indolent, and small, well-circumscribed lesions may clinically and radiologically mimic benign tumors [48]. Despite this indolent presentation, SS has a relatively high metastatic potential. This apparent paradox may be partly explained by *SS18*::*SSX*-driven epigenetic reprogramming, which may promote tumor cell plasticity and early dissemination. In addition, a low tumor mutational burden (TMB) and a “cold” tumor microenvironment (TME) may contribute to immune evasion and disease progression. SS of the GI tract may present as a polypoid or ulcerated mucosal lesion or as a transmural mass [32]. Patients with intrathoracic SS commonly report chest pain, dyspnea, cough, or hemoptysis, although some tumors are detected incidentally [49]. Renal SS typically presents with abdominal pain and hematuria [37,38], whereas intraprostatic SS is often associated with lower urinary tract symptoms, including dysuria, urinary frequency, and nocturia [41]. SS of the head and neck region usually manifests as a deep-seated palpable mass that may be painful or tender [50]. Ocular and orbital SS often present insidiously with pain, proptosis, periorbital swelling, and diplopia [45,46].

Table 1 summarizes the unusual sites of synovial sarcoma that have been confirmed by molecular analysis.

## 5. Radiological Features

Radiological studies are valuable for suggesting a preoperative diagnosis of SS, largely because of the frequent presence of calcifications [51]. On plain radiographs, most tumors appear as round or oval, lobulated soft tissue masses of moderate density, typically located near large joints. The adjacent bone is usually preserved; however, periosteal reaction, superficial cortical erosion, or bone invasion is observed in approximately 15–20% of cases. Extensive bone destruction is uncommon and generally associated with long-standing, large, poorly differentiated tumors.

**Figure 1:**
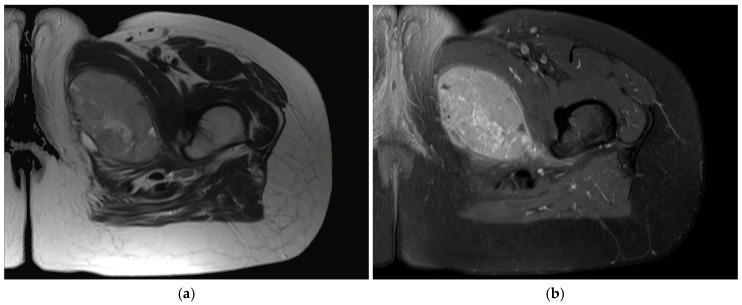
Magnetic resonance imaging (MRI) findings of synovial sarcoma. (**a**) Axial T2-weighted image shows a well-defined ovoid intramuscular mass in the left thigh with heterogeneous intermediate to high signal intensity. (**b**) Axial fat-suppressed contrast-enhanced T1-weighted image at the same level shows a well-circumscribed intramuscular mass with heterogeneous enhancement. All images are representative radiological and histopathological findings selected from the author’s institutional archives for illustrative purposes only. No new data analysis or previously unpublished data are included.

The most characteristic radiologic feature of SS, observed in approximately 15–20% of cases, is the presence of multiple small, spotty radiopacities resulting from focal calcification and, less commonly, from bone formation [52]. In most cases, these changes appear as fine stippling; however, larger radiopaque masses may occasionally partially or completely outline the tumor. Computed tomography (CT) and magnetic resonance imaging (MRI) are valuable for assessing the site of origin and extent of disease, typically demonstrating a para-articular, heterogeneous, septated mass with calcification or focal bone erosion, consistent with findings on conventional radiographs (Figure 1) [53,54]. Dynamic contrast-enhanced MRI (DCE-MRI) provides functional information beyond conventional morphology by enabling quantitative assessment of tumor vascularity and perfusion. In SS, it may aid in lesion characterization, early assessment of response to neoadjuvant therapy, and differentiation from other soft-tissue tumors with overlapping imaging features [55].

## 6. Molecular Features

SS is defined by a distinctive molecular profile dominated by a recurrent, disease-defining chromosomal translocation. However, no established predisposing factors have been identified, although rare cases have been reported following radiotherapy [56]. Molecularly, SS is characterized by a pathognomonic t(X;18)(p11;q11), which fuses *SS18* on chromosome 18 with one of the *SSX* genes (*SSX1*, *SSX2*, or *SSX4*) on the X chromosome [57,58]. The most common rearrangement involves fusion of exon 10 of *SS18* with exon 6 of the partner *SSX* gene [59], generating the oncogenic *SS18*::*SSX1/2/4* fusion [60].

The central oncogenic driver in SS is the *SS18*::*SSX* fusion, which is essential for maintaining the transformed phenotype [61]. The conditional expression of *SS18*::*SSX* induces SS in genetically engineered mouse models, supporting the oncogenic role of the fusion in permissive mesenchymal progenitor cells [62,63]. Furthermore, *SS18*::*SSX1* has been shown to transform primary cell lines [64]. At the molecular level, the fusion protein disrupts epigenetic regulation and impairs mesenchymal differentiation through several mechanisms. These include displacement of native SS18 from the canonical SWI/SNF chromatin-remodeling complex [65], induction of dependence on BRD9-containing non-canonical SWI/SNF (ncBAF) complexes [66,67], repression of activating transcription factor 2 (ATF2) target genes through interaction with ATF2 and TLE1 [68], and recruitment of the KDM2B lysine demethylase to unmethylated CpG islands [69,70].

Approximately two-thirds of SSs harbor an *SS18*::*SSX1* fusion and one-third an *SS18*::*SSX2* fusion, whereas *SS18*::*SSX4* and the rare *SS18L1*::*SSX1* fusion arising from t(X;20) are uncommon [58,59,71]. Tumors with *SS18*::*SSX2* are almost exclusively monophasic, whereas those with *SS18*::*SSX1* exhibit an approximate 2:1 ratio of monophasic to biphasic morphology [62,72]. *SS18*::*SSX1*-positive tumors show an approximately equal sex distribution, whereas *SS18*::*SSX2*-positive tumors are more frequent in women [57,62].

A small subset of *SS18*-negative tumors has been reported to harbor distinct *SSX1* fusions, including *SS18L1*::*SSX1*, *EWSR1*::*SSX1*, and *MN1*::*SSX1* [73]. In addition, a novel *SS18*::*NEDD4* gene fusion has been identified in a primary renal SS [74]. In pediatric SS, the mutational burden is particularly low compared with both adult SS and other sarcomas, further underscoring the fusion-driven biology of this tumor [75,76]. In a small subset of cases, potentially targetable *BRAF* p.V600E mutations have also been identified [77]. Due to their low prevalence (3–5%), routine screening is not recommended in SS; however, selective testing may be considered in advanced or treatment-refractory cases, or when alternative *BRAF*-mutant tumors are suspected.

## 7. Histopathological Features

### 7.1. Macroscopic Features

**Figure 2:**
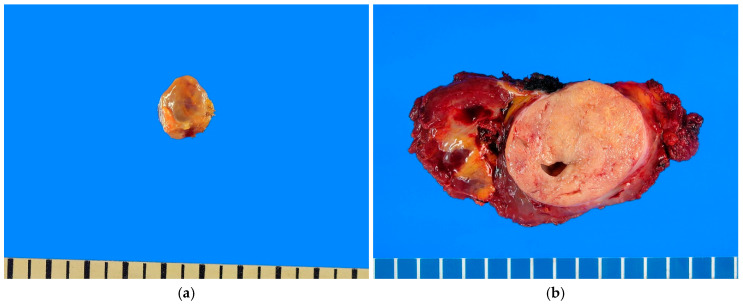
Macroscopic findings of synovial sarcoma. (**a**) The cut surface shows a well-circumscribed grayish-brown to tan nodule measuring 1.0 × 1.0 cm. (**b**) The tumor is well circumscribed, gray to white, and predominantly solid with focal cystic changes, measuring 6.5 × 5.5 cm. All images are representative radiological and histopathological findings selected from the author’s institutional archives for illustrative purposes only. No new data analysis or previously unpublished data are included.

Most SSs measure approximately 3–10 cm at diagnosis, although minute lesions (<1 cm) may occur, particularly in the hands and feet [78]. On gross examination, the color and consistency of the tumor vary according to histological composition and may range from tan to pink and from soft to firm; slow-growing lesions are usually well circumscribed (Figure 2). SS is often multinodular and may show cystic change. Meanwhile, calcification, metaplastic ossification, or necrosis may also be observed.

The gross appearance of SS varies by anatomic site. In the esophagus, SS most commonly presents as a polypoid mucosal mass; meanwhile, in the stomach, early lesions typically appear as small (1–2 cm), cup- or plaque-like mucosal lesions, or as subserosal masses. Mucosal tumors often extend into the submucosa and frequently involve the muscularis propria. With progression, tumors may form transmural masses measuring approximately 5–10 cm. Intrathoracic SS usually presents as a large, well-circumscribed mass, often with cystic change, calcification, or necrosis. Renal SS typically presents as a large, tan, rubbery mass with cystic change, hemorrhage, and necrosis, and may extend into adjacent structures, such as the renal vein, renal hilum, and perirenal adipose tissue [40]. Intraprostatic SS exhibits gross features similar to those of renal SS.

### 7.2. Histopathology

SS has a broad histological spectrum and is subdivided into monophasic, biphasic, and poorly differentiated subtypes. In some cases, all three subtypes may coexist within a single tumor. The most common subtype is monophasic SS, followed by biphasic SS, which contains epithelial elements, usually glandular, and spindle cells. Poorly differentiated SS refers to tumors with an undifferentiated, high-grade appearance.

SSs exhibit site-dependent histological variability. In the GI tract, most cases are low-grade with few mitoses, although high-grade features may occur and are associated with a poorer prognosis. Thoracic monophasic SS consists of monomorphic spindle cells, and the presence of entrapped pneumocytes can complicate the diagnosis. In the genitourinary tracts, monophasic SS features monomorphic blue spindle cells, frequently with hemangiopericytoma-like vessels and dystrophic calcification. Poorly differentiated SS in these areas reflects more aggressive behavior, with high mitotic activity and nuclear atypia. Head and neck SS may present as monophasic or biphasic tumors. Tumors with a predominance of epithelial cells can closely resemble adenocarcinoma, and poorly differentiated tumors exhibit frequent mitotic activity and necrosis.

#### 7.2.1. Monophasic Synovial Sarcoma

**Figure 3:**
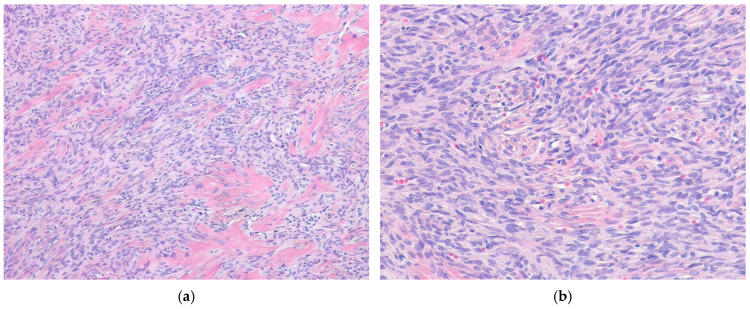
Monophasic synovial sarcoma. (**a**) The tumor is composed of relatively uniform spindle cells arranged in a fascicular pattern, with wiry collagen fibers and focally thicker collagen bundles (H&E stain, ×100). (**b**) At higher magnification, the tumor cells show elongated vesicular nuclei with fine chromatin and scant cytoplasm (H&E stain, ×200). All images are representative radiological and histopathological findings selected from the author’s institutional archives for illustrative purposes only. No new data analysis or previously unpublished data are included.

The monophasic SS subtype accounts for approximately 50–60% of cases. Most monophasic tumors are composed of spindle cells, although some authors also recognize a rare monophasic epithelial subtype [79,80,81]. These monophasic spindle cells are relatively small and delicate, exhibiting a fairly uniform morphology (Figure 3). Indeed, these cells have ovoid, hyperchromatic nuclei with fine granular chromatin and inconspicuous nucleoli, scant basophilic cytoplasm, and indistinct cell borders. The nuclear-to-cytoplasmic (N:C) ratio is usually high, often resulting in apparent nuclear overlap. Architecturally, the spindle cells are typically arranged in densely cellular sheets or fascicles, and nuclear palisading or a herringbone pattern may occasionally be present. The amount of collagen is variable and usually scant; however, ropy or wiry collagen strands, hyalinized collagen bands, or areas of dense fibrosis may be present. Cellular clusters with a vague epithelioid appearance may be interspersed among the spindle cells, and nuclear pleomorphism is typically absent. Mitotic activity is variable, and tumor necrosis may be present. A subset of monophasic SS may harbor foci of poorly differentiated (round cell) morphology. Focal myxoid change may be present [82]. Furthermore, monophasic SS is often associated with alternating hypocellular and hypercellular areas, as well as retiform cords or microcysts. Many SSs commonly display a hemangiopericytoma-like (staghorn-shaped) vascular pattern, and mast cells are variably present. Perivascular hyalinization may be present. Calcification and/or ossification are observed in up to one-third of cases and may be extensive. In areas of ossification, lace-like osteoid formation mimicking osteosarcoma may be observed, and the bone may subsequently mature to a lamellar or trabecular architecture [83]. Metaplastic cartilage is rarely identified. Myoid and neuroendocrine differentiation in SSs has also been reported in rare cases [84,85,86].

#### 7.2.2. Biphasic Synovial Sarcoma

**Figure 4:**
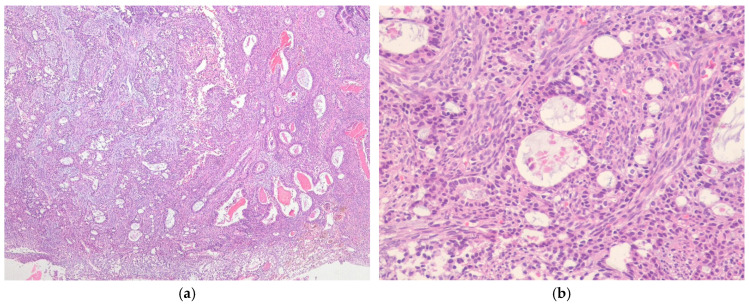
Biphasic synovial sarcoma. (**a**) The tumor shows a biphasic pattern composed of glandular epithelial components and spindle cell components (H&E stain, ×40). (**b**) The spindle cell component resembles that of monophasic synovial sarcoma, while the glandular lumina contain mucin and eosinophilic secretions (H&E stain, ×200). All images are representative radiological and histopathological findings selected from the author’s institutional archives for illustrative purposes only. No new data analysis or previously unpublished data are included.

Biphasic SS accounts for approximately 20–30% of cases and is characterized by the coexistence of epithelial and spindle cell components in variable proportions (Figure 4). The epithelial cells are arranged in solid nests or cords and frequently form glandular structures, most often with tubular architecture, and less commonly, alveolar or papillary patterns. In glandular areas, the epithelial cells are cuboidal or columnar, with ovoid vesicular nuclei and relatively abundant, pale eosinophilic cytoplasm compared with the basophilic spindle cell component. Glandular lumina may contain epithelial mucin. Some biphasic tumors contain an extensive epithelial component, which appears as paler or more eosinophilic areas; however, the glandular differentiation is often rudimentary. Rarely, the epithelial cells may exhibit keratinizing squamous metaplasia or granular cell change [87]. The spindle cells in biphasic SS are composed of uniform, mildly hyperchromatic spindle cells that closely resemble those observed in monophasic SS.

#### 7.2.3. Poorly Differentiated Synovial Sarcoma

**Figure 5:**
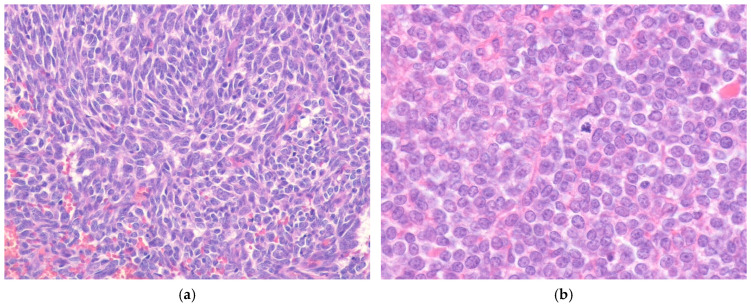
Poorly differentiated synovial sarcoma. (**a**) The tumor is hypercellular and composed of spindle-shaped cells with nuclear atypia (H&E stain, ×200). (**b**) The tumor shows a proliferation of relatively uniform round cells with mitotic figures (H&E stain, ×200). All images are representative radiological and histopathological findings selected from the author’s institutional archives for illustrative purposes only. No new data analysis or previously unpublished data are included.

Poorly differentiated SS accounts for approximately 10–15% of all cases and is characterized by increased cellularity, greater nuclear atypia, and high mitotic activity (>6 mitoses/mm^2^ or >10 mitoses per 10 high-power fields [HPFs] of 0.17 mm^2^) [88]. Poorly differentiated areas may be composed of fascicular spindle cells (resembling high-grade fibrosarcoma or malignant peripheral nerve sheath tumor (MPNST)), small round hyperchromatic tumor cells (resembling Ewing sarcoma), or epithelioid cells (Figure 5) [89]. Compared with conventional biphasic or monophasic SS, poorly differentiated areas more often exhibit necrosis, a branching vascular pattern, and thin fibrovascular septa that separate nests or clusters of tumor cells [90]. Rhabdoid cells, characterized by perinuclear cytoplasmic inclusions composed of intermediate filaments, may occasionally be present. These tumors can also contain a more differentiated biphasic or monophasic component. Poorly differentiated SS is rare in children [91], but these lesions are more common in older patients and frequently occur in metastatic SS. Poorly differentiated areas may consist of small, round hyperchromatic tumor cells (resembling small round blue cell tumors) or fascicular spindle cells (resembling MPNST).

#### 7.2.4. Myxoid Synovial Sarcoma

**Figure 6:**
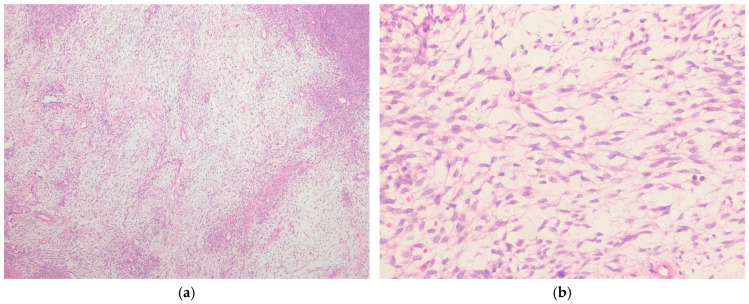
Myxoid synovial sarcoma. (**a**) The tumor shows extensive myxoid stromal change (H&E stain, ×40). (**b**) The tumor is hypocellular and composed of bland spindle cells in a myxoid stroma (H&E stain, ×200). All images are representative radiological and histopathological findings selected from the author’s institutional archives for illustrative purposes only. No new data analysis or previously unpublished data are included.

Myxoid SS is a rare subtype characterized by diffuse, prominent myxoid changes involving most of the tumor and exhibiting a hypocellular, bland histological appearance (Figure 6) [82,92,93]. Focal myxoid change is a well-recognized feature of SSs, but the presence of a predominantly myxoid stroma is rare.

#### 7.2.5. Calcifying/Ossifying Synovial Sarcoma

**Figure 7:**
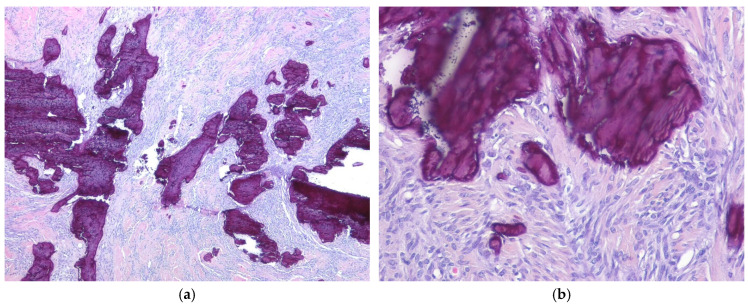
Calcifying/ossifying synovial sarcoma. (**a**) The tumor shows extensive stromal calcification (H&E stain, ×40). (**b**) The tumor cells are uniform, spindle-shaped, and resemble those seen in monophasic synovial sarcoma (H&E stain, ×200). All images are representative radiological and histopathological findings selected from the author’s institutional archives for illustrative purposes only. No new data analysis or previously unpublished data are included.

Focal calcifications are common in SSs, whereas extensive calcification and ossification are uncommon (Figure 7) [52,94]. Approximately one-third of cases show calcification and/or ossification, which can occasionally be extensive. In areas of ossification, the osteoid exhibits a lace-like pattern that can mimic osteosarcoma, and the bone tissue may mature into lamellar or trabecular forms.

Table 2 summarizes the histological subtypes of SS, the associated histological and molecular features, and the major differential diagnoses.

## 8. Immunohistochemical Features

**Figure 8:**
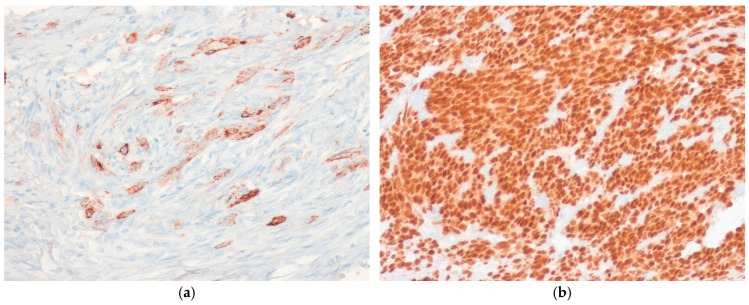
Immunohistochemical findings for synovial sarcoma. (**a**) The tumor cells show multifocal expression of cytokeratin (AE1/AE3) (immunohistochemical stain for cytokeratin [AE1/AE3], ×200). (**b**) The tumor cells show strong diffuse nuclear staining for SS18-SSX antibody (immunohistochemical stain for SS18-SSX antibody, ×200). All images are representative radiological and histopathological findings selected from the author’s institutional archives for illustrative purposes only. No new data analysis or previously unpublished data are included.

Most SSs express epithelial membrane antigen (EMA) and cytokeratins (CKs) [95,96], although the EMA is more consistently expressed than CKs, particularly in monophasic and poorly differentiated SS subtypes (Figure 8a). Transducin-like enhancer of split 1 (TLE1) shows characteristic nuclear immunoreactivity in up to 95% of cases [97,98]; however, this protein lacks specificity and may also be expressed in histological mimics, including MPNST and solitary fibrous tumor (SFT) [99,100,101,102]. Recently, antibodies have been developed that are specific to the *SS18*::*SSX* fusion protein (clone E9X9V) and to the SSX C-terminus (clone E5A2C), which provide strong, diffuse nuclear staining and show excellent diagnostic performance, with sensitivity and specificity exceeding 95% for SS (Figure 8b) [103,104,105].

Smooth muscle actin (SMA) and S100 protein expression are variable, whereas desmin and CD34 are only rarely expressed [96]. Furthermore, CD56 is frequently positive [36], synaptophysin is occasionally expressed [86], and calretinin expression is common [106]. Positivity for DOG1 and, less commonly, focal KIT expression may also be observed. Most SSs express B-cell lymphoma 2 (BCL2) and CD99, which may show membranous staining [107]. Loss of SMARCB1 (INI1) and H3K27me3 expression, either partially or completely, has been reported in a subset of SS [108,109,110,111]. Trichorhinophalangeal syndrome type 1 (TRPS1), a recently identified biomarker for breast carcinoma, is also frequently expressed in SS [112,113]. TRPS1 expression is associated with the activity of the *SS18*::*SSX* fusion oncoprotein and may help avoid diagnostic pitfalls in tumor identification. Cancer-testis antigens, including NY-ESO-1, PRAME, MAGEA1, and MAGEA4, are frequently expressed in SS, supporting the potential use of these antigens as targets for immunotherapy [114].

Table 3 summarizes the immunohistochemical profile of SS and the associated diagnostic implications.

## 9. Diagnostic Approach

A systematic diagnostic approach is crucial for the accurate diagnosis of SS. The first step involves careful histological examination of hematoxylin and eosin (H&E)-stained sections at low magnification, with particular attention to tumor cell morphology, architectural patterns, and stromal features. Extensive sampling is strongly recommended, as this method may more effectively facilitate the identification of key diagnostic features, particularly glandular differentiation, than ancillary studies [115]. In rare cases, the glandular component may predominate and mimic adenocarcinoma; however, at least a spindle cell component is almost always identifiable. Furthermore, differentiated components may be observed in other areas of tumors that are otherwise poorly differentiated. Therefore, correlating clinical history and radiologic findings is critical for distinguishing SS from histological mimics.

The differential diagnosis of SS is broad and includes several morphological mimics, such as fibrosarcomatous dermatofibrosarcoma protuberans (DFSP), SFT, adult fibrosarcoma, Kaposi sarcoma, leiomyosarcoma, alveolar RMS, spindle cell/sclerosing RMS, GI stromal tumor, MPNST, neurotrophic tyrosine receptor kinase (NTRK)-rearranged spindle cell neoplasm, Ewing sarcoma, sarcomas with *BCOR* genetic alterations (e.g., *BCOR*-rearranged sarcoma), biphenotypic sinonasal sarcoma, and malignant mesothelioma (sarcomatoid). Table 4 summarizes the differential diagnoses of SS, highlighting the key clinical, histological, immunohistochemical, and molecular features. Clinicians and pathologists should also be aware that small lesions can be misinterpreted as benign processes, potentially leading to diagnostic delay.

**Figure 9:**
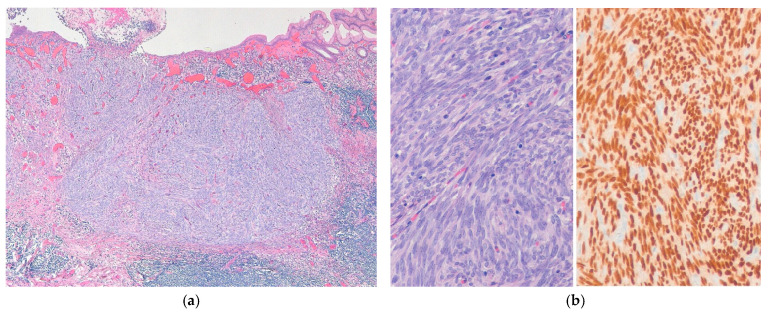
Primary monophasic synovial sarcoma of the stomach. (**a**) Microscopic examination shows a relatively well-circumscribed, spindle cell lesion beneath the gastric mucosa (H&E stain, ×40). (**b**) At high magnification, the lesion is composed of fascicles of monomorphic spindle cells with elongated nuclei and scant cytoplasm (left) (H&E stain, ×200). Immunohistochemistry demonstrates strong diffuse nuclear expression for SS18–SSX, supporting the diagnosis of synovial sarcoma (right) (immunohistochemical stain for SS18-SSX antibody, ×200). All images are representative radiological and histopathological findings selected from the author’s institutional archives for illustrative purposes only. No new data analysis or previously unpublished data are included.

A targeted immunohistochemical panel is essential for evaluating and classifying soft tissue tumors. Immunohistochemistry (IHC) using an *SS18*::*SSX* fusion-specific antibody, together with a C-terminus SSX-specific antibody, is a valuable first-line diagnostic tool. This approach is particularly helpful in diagnostically challenging cases occurring at unusual anatomical sites (Figure 9). In particular, the differential diagnosis with MPNST warrants careful consideration, as monophasic SS characteristically displays more uniform nuclei with smooth contours, lacking the wavy or buckled nuclei typical of MPNST. Immunohistochemically, the *SS18*::*SSX* fusion-specific antibody shows strong nuclear positivity in SS; in contrast, MPNST typically shows S100 protein and SOX10 positivity and may exhibit loss of H3K27me3. Although IHC provides important diagnostic information, its results should be interpreted in conjunction with clinical and histopathological findings to ensure diagnostic accuracy. When histological and IHC findings are inconclusive, molecular testing for the *SS18*::*SSX* fusion is recommended [116]. Thus, molecular testing has become increasingly important in diagnosing soft tissue tumors. Ultimately, rigorous diagnostic criteria must always be applied.

## 10. Treatment

SS is primarily managed with surgical resection, often combined with radiotherapy for local control, while systemic chemotherapy is reserved for high-risk, advanced, or metastatic disease. Despite these multimodal approaches, outcomes in patients with metastatic disease remain suboptimal. Accordingly, novel strategies, including molecularly targeted agents, immunotherapy, and epigenetic-based therapeutic approaches, are under active investigation [117,118,119,120,121].

### 10.1. Surgical Excision

SS is frequently misdiagnosed due to the associated atypical presentation, leading to unplanned excisions that increase the risk of recurrence and metastasis [122]. Surgical excision with negative margins remains the cornerstone of SS treatment, in accordance with established soft-tissue sarcoma guidelines [123]. The primary goal of limb salvage surgery is to achieve oncologically adequate margins while preserving limb function. For small (<5 cm), superficial tumors distant from critical structures, wide excisions with 1–2 cm negative margins are generally sufficient [124]. For tumors located near vital structures, margins defined by the epineurium, adventitia, or periosteum may help preserve function but often result in close or positive margins, necessitating adjuvant radiotherapy to reduce the risk of local recurrence [125,126].

For isolated locoregional recurrence, surgical re-excision with negative margins is preferred; adjuncts such as re-irradiation or isolated limb perfusion may facilitate limb salvage, while amputation is reserved for unresectable disease. In metastatic synovial sarcoma, resection of selected oligometastatic disease, particularly in the lungs, may be considered, with treatment decisions guided by disease-free interval, tumor burden, performance status, and multidisciplinary evaluation.

Although regional lymph node metastases are uncommon in synovial sarcoma, they are associated with a worse prognosis. Suspicious nodes should be evaluated with imaging and, where feasible, pathologic confirmation; routine elective nodal treatment is not recommended, but confirmed nodal disease should be managed within a multidisciplinary framework.

### 10.2. Radiation Therapy

Radiation therapy is a crucial component of SS management, particularly for tumors larger than 5 cm or when surgical margins are limited by proximity to critical structures [123]. The primary role of radiation therapy is to enhance local control, potentially improving overall survival [127]. The decision between neoadjuvant and adjuvant radiotherapy depends on patient- and tumor-specific factors [128]. Neoadjuvant radiotherapy may facilitate limb salvage surgery by reducing tumor size, but this method is associated with a higher risk of wound complications. In contrast, adjuvant radiotherapy targets residual microscopic disease yet may cause late toxicities, such as fibrosis and joint stiffness. Advances in radiation techniques, particularly intensity-modulated radiation therapy, enable more precise, targeted dose delivery while sparing surrounding normal tissues, making this method especially suitable for preoperative treatment [129,130]. Radiotherapy can also be used as a definitive or palliative modality to achieve disease control and improve quality of life in patients with advanced disease or significant comorbidities who are not candidates for surgery.

Adjuvant external beam radiotherapy (EBRT) (50–66 Gy) is recommended for SS ≥ 5 cm or with close/positive margins, improving local recurrence-free survival [128]. Neoadjuvant EBRT (50 Gy in 25 fractions) can enable limb-sparing surgery in borderline-resectable tumors but carries a higher risk of wound complications compared with postoperative treatment [129,130].

### 10.3. Chemotherapy

Chemotherapy plays a crucial role in the management of SS, particularly in advanced disease where local treatments are insufficient. Anthracycline-based regimens remain the standard first-line systemic therapy [131,132], with combination therapy showing superior efficacy [133,134]. Chemotherapeutic responsiveness varies by age and risk group. In pediatric and adolescent patients with high-risk disease, doxorubicin–ifosfamide-based regimens are commonly used in the neoadjuvant or adjuvant setting to improve long-term outcomes [135,136]. In adults, chemotherapy is more individualized and tailored to tumor characteristics, disease extent, and patient-related factors, including treatment tolerance [137].

Alternative agents have been explored for patients with disease refractory to standard regimens. Gemcitabine-based combinations have shown limited efficacy in SS and are not favored in later-line settings [138,139]. Trabectedin, approved for advanced soft tissue sarcoma after anthracycline failure, has demonstrated activity in SS, possibly by disrupting fusion-driven oncogenic transcription. Therefore, chemotherapy selection requires a careful balance between the expected clinical benefit and the associated treatment-related toxicity [140,141].

In the perioperative setting, neoadjuvant chemotherapy—most commonly anthracycline–ifosfamide-based—may be considered in selected patients with large, deep-seated, or borderline resectable high-risk SS, although supporting evidence remains limited and its use should be individualized. Postoperative radiotherapy is the primary modality for local control in tumors with high-risk features, whereas adjuvant chemotherapy is reserved for carefully selected high-risk patients without a uniform indication. All perioperative treatment decisions should be guided by multidisciplinary evaluation and individualized risk stratification.

### 10.4. Tyrosine Kinase Inhibitors

Tyrosine kinase inhibitors (TKIs) represent important targeted therapies for advanced SS, particularly in metastatic or unresectable disease. Pazopanib, a multitarget TKI and the only approved targeted agent for non-adipocytic metastatic soft tissue sarcomas, has demonstrated improved progression-free survival (PFS) in phase III trial and activity in treatment-refractory SS, although a consistent overall survival benefit has not been established [142,143,144,145]. Regorafenib has also demonstrated activity in soft tissue sarcomas, with randomized trials showing improved PFS compared with placebo, and updated analyses support the use of regorafenib in SS as a later-line option [146]. By inhibiting VEGFR, PDGFR, and FGFR pathways implicated in SS biology, anlotinib may represent an additional second- or later-line therapeutic option [147].

### 10.5. Epigenetic Therapy

Epigenetic therapies have attracted considerable interest in SS because the *SS18*::*SSX* fusion protein disrupts chromatin regulation by altering interactions between the SWI/SNF and polycomb complexes, including loss of EZH2 repression by BAF47. Since EZH2 mediates transcriptional silencing through histone methylation, inhibiting EZH2 is a rational therapeutic strategy that may restore tumor suppressor gene expression [148]. Preclinical studies have demonstrated that EZH2 inhibition reduces SS cell proliferation and migration, further supporting EZH2 as a promising therapeutic target [149].

Histone acetylation represents another epigenetic vulnerability in SS. Histone deacetylase inhibitors promote chromatin relaxation and reactivation of silenced genes and have shown antitumor effects in preclinical models [150]. However, phase II trials of vorinostat and panobinostat in previously treated soft tissue sarcoma cohorts, including SS, failed to demonstrate objective responses, limiting their current clinical role [151,152].

Bromodomain (BRD) inhibition offers an additional therapeutic strategy by disrupting acetylation-dependent transcriptional programs. In particular, BRD9 has emerged as a promising target in SS; however, BRD9 degraders (e.g., FHD-609 [NCT04965753]; CFT8634 [NCT05355753]) have revealed limitations, including insufficient single-agent efficacy and cardiac toxicity, highlighting the need for combination strategies and safer next-generation compounds [67]. Overall, therapies targeting *SS18::SSX*-driven chromatin dysregulation provide a biologically rational approach in SS; however, clinical efficacy remains modest, and further investigation—particularly of combination strategies—is warranted.

### 10.6. Immunotherapy

Immunotherapy has shown limited efficacy in most soft tissue sarcomas. Immune checkpoint inhibitors (ICIs), including pembrolizumab, nivolumab, and ipilimumab, have demonstrated limited efficacy in SS [153,154]. This limited response is attributed to the “cold” TME and low TMB. In contrast, engineered T cell receptor (TCR) therapies enable human leukocyte antigen (HLA)-restricted recognition of intracellular cancer-testis antigens. This approach represents a shift from nonspecific checkpoint blockade to antigen-specific adoptive cellular therapy in SS [155].

NY-ESO-1, a cancer-testis antigen that is highly expressed in many SS cases, is one of the most promising immunotherapy targets. Similarly, MAGE-A4-directed TCR therapy has shown meaningful clinical activity. Afamitresgene autoleucel (afami-cel), an HLA-restricted engineered TCR therapy targeting MAGE-A4, has demonstrated durable responses in heavily pretreated patients with MAGE-A4-expressing SS [156]. Combination approaches with ICIs are under investigation to enhance T cell persistence and improve antitumor efficacy [157,158].

### 10.7. Other Emerging Targeted Therapies

Several additional targeted strategies are under investigation in SS. High-throughput siRNA screening has identified a critical dependency of SS cells on the DNA damage response kinase ataxia telangiectasia and Rad3-related protein (ATR) [159]. In preclinical models, the ATR inhibitor berzosertib (VX970/VE-822) induced synthetic lethality and suppressed tumor growth in patient-derived xenografts [160]. This vulnerability is associated with *SS18*::*SSX* fusion expression, increased γH2AX levels, and apoptosis. Meanwhile, combination strategies with cisplatin or PARP inhibitors have further enhanced antitumor effects, supporting ATR inhibition as a potential therapeutic strategy [160].

Cyclin-dependent kinases (CDKs) are frequently dysregulated in cancer [161,162]; thus, as key regulators of cell cycle progression and transcription, CDKs represent another promising target in SS. Preclinical studies have shown that inhibition of CDK4/6, CDK7, and CDK9 suppresses SS cell proliferation by disrupting cell cycle control and transcriptional programs [163,164,165]. Although palbociclib, a selective CDK4/6 inhibitor, has shown preliminary activity in SS, its clinical benefits remain to be established [166].

The insulin-like growth factor-1 receptor (IGF-1R) pathway has been implicated in SS pathogenesis, with IGF-1R overexpression associated with tumor cell proliferation and survival [167]. The *SS18*::*SSX* fusion protein has been reported to upregulate IGF2 via epigenetic mechanisms, potentially establishing an autocrine/paracrine growth loop [168]. Early-phase clinical trials of IGF-1R inhibitors (figitumumab and cixutumumab) have demonstrated modest activity predominantly in Ewing sarcoma, with limited efficacy observed in SS [169,170]. Combination strategies are under investigation.

Table 5 summarizes the systemic treatment strategies for SS, including drug classes, mechanisms of action, and clinical settings.

## 11. Prognosis

SS shows a variable clinical course, with approximately 40% of patients developing metastatic disease. Metastases most commonly involve the lungs, and less frequently, regional lymph nodes. Although most recurrences occur within the first few years after diagnosis, late recurrences beyond 10 years are well recognized [171]. Prognosis is primarily determined by tumor stage at presentation, tumor size, and Fédération Nationale des Centres de Lutte Contre le Cancer (FNCLCC) histological grade, patient age, and primary site [172]. Tumors > 5 cm, non-extremity location, advanced stage, and higher FNCLCC grade are associated with adverse outcomes. The presence of >20% poorly differentiated components is associated with aggressive behavior [173]. Conversely, tumors <5 cm with low mitotic activity (<6 mitoses/mm^2^, equivalent to <10 mitoses/1.7 mm^2^) and absence of necrosis are associated with favorable outcomes [89]. Minute SSs (<1 cm) have an excellent prognosis.

Children demonstrate superior survival compared with adults. Population-based studies report 5- and 10-year disease-specific survival rates of approximately 76–90% and 65–75% in pediatric patients, compared with 62% and 52% in adults, respectively [174,175]. Extremity-based tumors are associated with more favorable outcomes than tumors arising in the trunk or the head and neck region. Although the prognostic significance of *SS18*::*SSX1/2/4* fusion types has been investigated, the results have been inconsistent [176,177]. Therefore, fusion subtypes are not considered reliable prognostic markers. Higher genomic complexity has been associated with increased metastatic potential [76]. Intrathoracic SS is associated with poorer outcomes compared with conventional soft tissue SS [34,178,179]. Similarly, renal and intraprostatic SS are linked to adverse outcomes and frequently present with pulmonary and other distant metastases.

After curative treatment for SS, surveillance should be risk-adapted, taking into account tumor size, depth, site, FNCLCC grade, histological features, and surgical margin status. Nomogram-based tools (SARCULATOR and PERSARC) can help with individualized risk stratification. According to the 2021 ESMO–EURACAN–GENTURIS guidelines [137], high-risk patients should undergo a chest CT and a local MRI every 3–4 months for the first 2–3 years, then every 6 months, and finally annually. Given that pulmonary metastasis is the predominant site of distant relapse in SS and that there is a well-established propensity for late recurrence, chest imaging remains central, and surveillance should continue beyond 10 years, regardless of risk category.

## 12. Future Perspectives

The *SS18*::*SSX* fusion is pathognomonic for SS and serves as a robust diagnostic hallmark; however, the prognostic significance of this fusion remains incompletely defined. Despite significant progress in elucidating the molecular and epigenetic mechanisms underlying SS—particularly the role of *SS18*::*SSX* in chromatin remodeling and transcriptional reprogramming—therapeutic advances have been modest. The development and validation of predictive biomarkers to support refined prognostic stratification and biomarker-driven therapeutic decision-making remain critical needs in SS [157].

Recent experimental data support a cell-of-origin model. Hill et al. [180] demonstrated that SS arises from a rare *Hic1*^+^ *Pdgfra*^+^ *Lgr5*^+^ fibroblastic subset, suggesting that a permissive epigenomic landscape is required for *SS18*::*SSX*-driven oncogenic transformation. However, because these findings are primarily based on murine models, validation in well-annotated human cohorts is necessary to confirm the translational relevance. In parallel, many emerging therapeutic strategies remain in preclinical or early-phase development, with limited long-term efficacy data and challenges in patient recruitment [181]. Therefore, clear validation in human studies and the development of biomarker-driven therapeutic strategies will be necessary to translate mechanistic insights into sustained clinical benefit for patients with SS.

DNA methylation profiling may serve as an ancillary tool in diagnostically challenging cases where morphology, immunophenotype, and conventional molecular testing are inconclusive; however, its routine use remains limited by small reference cohorts and lack of prospective validation. Larger, multi-institutional synovial sarcoma–specific datasets and prospective studies are needed to establish its clinical utility and integration into diagnostic algorithms.

Prognostic nomograms such as SARCULATOR and PERSARC represent useful but imperfect decision-support tools for perioperative chemotherapy selection in localized synovial sarcoma; further refinement incorporating molecular biomarkers, imaging parameters, and prospective validation across anatomical sites and rare soft tissue sarcoma subtypes will be essential to improve their clinical reliability and broader applicability.

## 13. Conclusions

SS is an aggressive mesenchymal malignancy with variable epithelial differentiation, defined by a pathognomonic *SS18*::*SSX1/2/4* fusion gene. Owing to the morphological overlap of SS with other soft tissue tumors, accurate diagnosis requires an integrated histopathological, immunohistochemical, and molecular evaluation. Although local control has improved with contemporary multimodality management, outcomes for patients with advanced or metastatic disease remain poor. Future progress will depend on translating molecular and epigenetic insights into biomarker-driven therapeutic strategies and precision clinical trials for SS.

## Figures and Tables

**Table 1:** Unusual sites of synovial sarcoma occurrence with molecular confirmation.

Sites	Notes	References
Bone	Predominantly involves the long bones of the extremities, with metaphyseal predilection	[28]
Central nervous system	Predominantly involves extra-axial (dural-based); rare intra-axial involvement; historically misdiagnosed as hemangiopericytoma or meningioma	[24]
Gastrointestinal tract	Predominantly involves the stomach; occasional cases in the esophagus, small intestine, and colon	[32]
Female genital tract	Predominantly involves the vulva and vagina	[31]
Head and neck	Parapharyngeal space, oropharynx, hypopharynx, larynx; typically affects young adults; slight male predominance	[42,43,44]
Heart/mediastinum	Extremely rare; associated with poor prognosis	[33,34,35]
Lung/pleura	Predominantly involves the pleura or lung parenchyma (>80% of intrathoracic cases); peak incidence in the 4th–5th decades	[34,36]
Ocular adnexa/orbit	Epibulbar region, anterior orbit, or intraconal space; female predominance; may be secondarily involved by metastatic synovial sarcoma	[45,46,47]
Peripheral nerve	Predilection for major peripheral nerves (e.g., ulnar, median, peroneal, and sciatic nerves); relatively favorable prognosis, likely related to smaller tumor size	[27]
Retroperitoneum/pelvic cavity/intra-abdomen	Often presents as a large mass; associated with a high local recurrence rate; pelvic tumors frequently metastasize to distant sites	[25]
Skin	Primary cutaneous synovial sarcoma is extremely rare; must be distinguished from cutaneous metastases	[29]
Urinary and male genital tract	Predominantly involves the kidney and prostate	[37,38,39,40,41]

**Table 2:** Histological subtypes of synovial sarcoma and their associated histological and molecular features, and major differential diagnoses.

Subtype	Incidence	Histological Features	Cytogenetic Alterations	Gene Fusion	Differential Diagnoses
Monophasic	50–60%	Densely cellular sheets or vague fascicles of short spindle cells with relatively uniform, ovoid nuclei that are often overlapping; lack of significant pleomorphism; highly variable mitotic activity (from sparse to numerous); variable collagenous stroma; calcification and metaplastic bone formation may be present; hemangiopericytoma-like (“staghorn”) vascular pattern may be present; necrosis is uncommon; rarely, purely glandular monophasic SS (termed monophasic epithelial SS) ^a^	t(X;18)(p11;q11) t(X;20)(p11;q13) t(X;22)(p11;q12)	*SS18*::*SSX1/2/4* ^b^ *SS18L1*::*SSX1* *EWSR1*::*SSX1* *MN1*::*SSX1*	Fibrosarcomatous DFSP Solitary fibrous tumor Adult fibrosarcoma Kaposi sarcoma Leiomyosarcoma Spindle cell/sclerosing rhabdomyosarcoma Gastrointestinal stromal tumor MPNST NTRK-rearranged spindle cell neoplasm Biphasic sinonasal sarcoma
Biphasic	20–30%	Epithelial and spindle cell components in varying proportions; epithelial cells with ovoid nuclei and abundant cytoplasm forming glands with lumina containing epithelial mucin, as well as papillary structures, nests, or cords; squamous metaplasia occurs in approximately 1% of cases; spindle cells are uniform and relatively small, with ovoid, pale-staining nuclei and scant cytoplasm	t(X;18)(p11;q11) t(X;20)(p11;q13) t(X;22)(p11;q12)	*SS18*::*SSX1/2/4* ^b^ *SS18L1*::*SSX1* *EWSR1*::*SSX1* *MN1*::*SSX1*	MPNST with glandular differentiation Carcinosarcoma Malignant mesothelioma Metastatic carcinoma
Poorly differentiated	10–15%	High cellularity, marked nuclear atypia, and increased mitotic activity (>6 mitoses/mm^2^ or >10 mitoses per 10 high-power fields (0.17 mm^2^)); fascicular spindle cells, small round hyperchromatic tumor cells, and epithelioid cells; tumor cells may be larger with more cytoplasm and may exhibit rhabdoid features	t(X;18)(p11;q11) (same as conventional SS)	*SS18*::*SSX* (same as conventional SS)	Alveolar rhabdomyosarcoma Ewing sarcoma *BCOR*::*CCNB3* (Ewing-like) sarcoma Malignant lymphoma Small cell carcinoma
Myxoid	<5%	Prominent myxoid change (>50% of the tumor area); foci of typical features of monophasic or biphasic SS	t(X;18)(p11;q11) (same as conventional SS)	*SS18*::*SSX* (same as conventional SS)	Myxoid sarcomas
Calcifying/ossifying	<5%	Extensive calcification and/or ossification; foci of typical features of monophasic or biphasic SS	t(X;18)(p11;q11) (same as conventional SS)	*SS18*::*SSX* (same as conventional SS)	Osteosarcoma

SS, synovial sarcoma; DFSP, dermatofibrosarcoma protuberans; MPNST, malignant peripheral nerve sheath tumor; NTRK, neurotrophic tyrosine receptor kinase. ^a^ Although monophasic epithelial SS may theoretically exist, this tumor is indistinguishable from adenocarcinoma without cytogenetic analysis. ^b^ *SS18::SSX1* is more frequently associated with biphasic morphology, whereas *SS18*::*SSX2* is strongly associated with monophasic tumors, and *SS18*::*SSX4* is rarely identified.

**Table 3:** Immunohistochemical profile of synovial sarcoma and the associated diagnostic significance.

Immunostaining	Approximate Positivity (%)	Comment
Fusion-related marker		
*SS18*::*SSX* fusion-specific antibody	>95	Strong, diffuse nuclear staining; high sensitivity and specificity for SS
SSX C-terminus-specific antibody	>95	Strong, diffuse nuclear staining; high sensitivity but less specificity for SS
Markers discovered by gene expression profiling		
TLE1 ^a^	~95	Moderate-to-strong nuclear staining ^a^
DOG1	10–30	Focal or weak expression
Epigenetic surrogate markers associated with *SS18*::*SSX* fusion		
H3K27me3	Loss	Partial or complete loss of H3K27me3 expression (~60% of cases)
Markers of aberrations in SWI/SNF chromatin-remodeling complex		
SMARCB1 (INI1)	Variable	Partial or reduced SMARCB1 (INI1) expression (70–90% of cases); complete loss is uncommon
Protein correlated with genetic alterations		
KIT (CD117)	5–10	Focal and weak expression
Epithelial markers		
CKs ^b^	>90	Focal expression in spindled cells; strong expression in epithelial components
EMA	>95	EMA expression is more frequent and more diffuse than CK expression
Neuroectodermal markers		
CD56	>90	Frequently expressed; may lead to misdiagnosis as small cell carcinoma
CD99	~70	Expression may be membranous or perimembranous, similar to Ewing sarcoma; lacks specificity
Synaptophysin	Rare	Rarely expressed; may lead to misdiagnosis as small cell carcinoma
Muscle markers		
SMA	20–40	Focal expression
Desmin	<5	Focal expression is rare
Endothelial markers		
CD34	Rare	Rare in monophasic SS; negative in poorly differentiated SS
Nerve sheath marker		
S100 protein	~40	Focal expression; may mimic nerve sheath tumors; potential diagnostic pitfall
SOX10	~40	Focal expression; may mimic nerve sheath tumors; potential diagnostic pitfall
Cancer-testis antigens		
NY-ESO-1	~60	High expression is significantly correlated with tumor necrosis and advanced clinical stage
PRAME	~85	
MAGEA1	~15	
MAGEA4 ^c^	~80	High expression is significantly correlated with tumor necrosis and advanced clinical stage
Others		
TRPS1	~85	Metastatic tumors more frequently exhibited strong and diffuse expression compared to primary tumors
BCL2	~90	Weak to strong staining; may have diagnostic utility in cases where EMA and CKs are negative
Calretinin	50–70	Frequently expressed; may lead to misdiagnosis as mesothelioma, particularly in pleural or pulmonary tumors

SSX, synovial sarcoma, X breakpoint; SS, synovial sarcoma; TLE1, transducin-like enhancer of split 1; DOG1, discovered on GIST-1; H3K27me3, trimethylation of histone H3 at lysine 27; SWI/SNF, Switch/sucrose non-fermentable; SMARCB1, SWI/SNF-related, matrix-associated, actin-dependent regulator of chromatin, subfamily B, member 1; INI1, integrase interactor 1; KIT, KIT proto-oncogene receptor tyrosine kinase; CK, cytokeratin; EMA, epithelial membrane antigen; SOX10, SRY-box transcription factor 10; NY-ESO-1, New York–esophageal squamous cell carcinoma-1; PRAME, preferentially expressed antigen in melanoma; MAGEA1, melanoma antigen gene A1; MAGEA4, melanoma antigen gene A4; TRPS1, trichorhinophalangeal syndrome type 1; BCL2, B-cell lymphoma 2. ^a^ TLE1 expression is not specific and can also be seen in histological mimics of SS, such as solitary fibrous tumor and malignant peripheral nerve sheath tumor. ^b^ Among CK subtypes, CK7 and CK19, as well as CK8/18 (CAM5.2), are most commonly expressed. ^c^ Co-expression of NY-ESO-1, PRAME, and MAGEA4 is significantly associated with adverse prognosis.

**Table 4:** Differential diagnoses of synovial sarcoma based on key clinical, histological, immunohistochemical, and molecular features.

Tumor Type	Clinical Features	Histological Features	Immunohistochemistry	Molecular Features
Fibrosarcomatous DFSP	Young to middle-aged adults; trunk, neck, back, shoulders	Spindle cells arranged in fascicular or herringbone pattern, cytologic atypia, elevated mitotic activity	Patchy or loss of CD34 expression	*COL1A1*::*PDGFB*
Solitary fibrous tumor	Adults (40–70 years); extremities, abdominal cavity, pelvis, retroperitoneum, head and neck	Ovoid spindled cells arranged in short or haphazard fascicles; branching, “staghorn” vasculature	CD34 (+), STAT6 (+)	*NAB2*::*STAT6*
Adult fibrosarcoma	Middle-aged and older adults; extremities, trunk, head and neck	Relatively monomorphic spindle cells with no nuclear pleomorphism; herringbone architecture	SMA (±), caldesmon (±)	*STRN*::*NTRK3*, *STRN3*::*NTRK3*
Kaposi sarcoma	Associated with HHV8 infection; skin, mucosal membranes, lymph nodes, visceral organs	Small slit-like vessels lined by mildly atypical endothelial cells with surrounding spindle cells	CD31 (+), CD34 (+), ERG (+), podoplanin (D2-40) (+), HHV8 (+)	Recurrent amplification of 11q13 (*FGF4*, *FGF3*), loss of Y chromosome
Leiomyosarcoma	Adults (usually 6th–7th decade); extremities, retroperitoneum, abdomen/pelvis, trunk	Intersecting fascicles of spindle-shaped tumor cells with elongated, blunt-ended (“cigar-shaped”) nuclei, nuclear atypia	SMA (+), desmin (+), caldesmon (+)	Mutation in *TP53*, *ATRX* alterations ^a^, *ALK* rearrangements (subset)
Alveolar rhabdomyosarcoma	Adolescents and young adults; extremities, head and neck, paraspinal, perineal regions	Primitive round cells with scant cytoplasm and hyperchromatic nuclei, alveolar pattern	Desmin (+), myogenin (+), MyoD1 (+)	*PAX3*::*FOXO1*, *PAX7*::*FOXO1*
Spindle cell/sclerosing rhabdomyosarcoma	Wide age ranges from infancy to adulthood; head and neck, paratesticular, visceral organs	Fascicles of spindle cells in intersecting or herringbone pattern; sclerosis or hyalinization; rhabdomyoblasts	Desmin (+), myogenin (+), MyoD1 (+)	*VGLL2*, *SRF*, *TEAD1*, *NCOA2*, or *CITED2* rearrangements, *MYOD1* p.Leu122Arg mutations
Gastrointestinal stromal tumor (GIST)	Wide age range (peak in 6th decade); gastrointestinal tract, mesentery, omentum, retroperitoneum	Broad morphological spectrum; spindle cell, epithelioid, or mixed morphology	KIT (CD117) (+); DOG1 (+); PDGFRA (+) ^b^, loss of SDHB expression ^c^	*KIT, PDGFRA* mutations, *SDH* mutations
Malignant peripheral nerve sheath tumor	Adults (20–50 years); rare in children, usually NF1-associated; trunk, extremities, head and neck	Spindle cells arranged in fascicles with alternating hypocellular and hypercellular areas; tapered or buckled nuclei	S100 protein (+) ^d^, SOX10 (+), loss of H3K27me3 expression	Mutations in *NF1*, *CDKN2A/CDKN2B*, PRC2 core components *(EED or SUZ12)*
NTRK-rearranged spindle cell neoplasm	Any age, predominantly pediatric; no sex predilection; extremities, trunk, head and neck	Haphazardly arranged spindled cells with infiltrative growth into adipose tissue, closely resembling lipofibromatosis	CD34 (+), S100 protein (+), pan-TRK (+)	*NTRK1*, *NTRK2*, and *NTRK3* rearrangements (e.g., *LMNA*::*NTRK1*, *TPR*::*NTRK1*, or *TPM3*::*NTRK1*)
Ewing sarcoma	Children and adolescents; long bone, pelvis, rib; ~12% extraskeletal	Uniform small round cells with finely dispersed chromatin and scant cytoplasm	CD99 (+), NKX2.2 (+), FLI1 (+), ERG (+) ^e^	*EWSR1*::*FLI1*, *EWSR1*::*ERG*, *EWSR1::FEV*, *EWSR1::ETV1*, *FUS::ERG*
Sarcoma with *BCOR* genetic alterations	Children and adolescents, with male predominance; pelvis, lower extremity, trunk, paraspinal region	Primitive round to spindle cells arranged in nests, sheets, or fascicles, with delicate vasculature	BCOR (+), CCNB3 (+), SATB2 (+), cyclin D1 (+), CD99 (+)	*BCOR::CCNB3*, *BCOR::MAML3*, *BCOR::ZC3H7B*
Biphenotypic sinonasal sarcoma	Adults (mean age, 53 years), marked female predominance; nasal cavity, ethmoid sinus	Fascicles of uniform spindle cells, hemangiopericytoma-like vasculature; rhabdomyoblastic elements (subset)	S100 protein (+), SMA (+), desmin (±), pan-TRK (+), β-catenin nuclear (+)	*PAX3::MAML3 (~60%)*, *PAX3::NCOA1*, *PAX3::NCOA2*, *PAX3::FOXO1*, *PAX3::WWTR1*
Malignant mesothelioma (sarcomatoid)	Adults (>50 years), with male predominance; pleura, peritoneum, pericardium, extrathoracic sites	Pleomorphic spindle cells forming fascicles; biphasic subtype may be present	Calretinin (+), CK5/6 (+), podoplanin (D2-40) (+), WT1 (+), loss of BAP1	*BAP1* mutations, *TRAF7* mutations, genomic near-haploidization

DFSP, dermatofibrosarcoma protuberans; STAT6, signal transducer and activator of transcription 6; SMA, smooth muscle actin; HHV8, human herpes virus 8; ERG, ETS-related gene; KIT, KIT proto-oncogene receptor tyrosine kinase; DOG1, discovered on GIST-1; PDGFRA, platelet-derived growth factor receptor alpha; SDHB, succinate dehydrogenase subunit B; NF1, neurofibromatosis type 1; H3K27me3, trimethylation of histone H3 at lysine 27; NTRK, neurotrophic tyrosine receptor kinase; pan-TRK, pan-tropomyosin receptor kinase; NKX2.2, NK2 homeobox 2; FLI1, Friend leukemia virus integration 1; BCOR, BCL6 corepressor; CCNB3, cyclin B3; SATB2, special AT-rich sequence-binding protein 2; WT1, Wilms tumor 1; BAP1, BRCA1-associated protein 1; +, positive staining; ±, variable staining. ^a^ *ATRX* alterations occur in 16–49% of leiomyosarcoma and likely contribute to the high frequency of alternative lengthening of telomeres. ^b^ *PDGFRA*-mutant GISTs usually demonstrate strong diffuse expression of PDGFRA. ^c^ SDH-deficient GISTs exhibit loss of SDHB protein expression irrespective of which *SDH* gene is mutated. ^d^ S100 protein is focally positive in 50–60% of cases. ^e^ FLI1 and ERG are often expressed in cases with the corresponding translocation.

**Table 5:** Systemic treatment strategies for synovial sarcoma.

Category	Agent	Mechanism of Action	Clinical Settings	References
Chemotherapy	Doxorubicin ± ifosfamide	DNA intercalation and topoisomerase II inhibition (doxorubicin); DNA alkylation (ifosfamide)	First-line for advanced or metastatic disease; neoadjuvant or adjuvant in selected high-risk patients	[131,132,133,134]
	Gemcitabine ± docetaxel	Antimetabolite and DNA synthesis inhibition (gemcitabine); microtubule stabilization and mitotic arrest (docetaxel)	Second-line or later-line; limited clinical efficacy	[138,139]
	Trabectedin	DNA minor groove binding; disruption of fusion-driven transcriptional programs	Second-line (post-anthracycline failure)	[140,141]
Tyrosine kinase inhibitor	Pazopanib	Multi-target TKI (VEGFR, PDGFR, FGFR)	Later-line for advanced or metastatic disease; improved PFS in phase III trials	[142,143,144,145]
	Regorafenib	Multi-target TKI (VEGFR1–3, PDGFR, FGFR, KIT, RAF1, TIE2)	Later-line for advanced or metastatic disease; improved PFS in randomized trials	[146]
	Anlotinib	Multi-target TKI (VEGFR, PDGFR, FGFR)	Second-line (post-anthracycline failure) or later-line; improved PFS in randomized trials	[147]
Epigenetic therapy	EZH2 inhibitors	Inhibit H3K27 trimethylation, restoring tumor suppressor gene expression	Investigational; preclinical suppression of proliferation and migration	[148,149]
	HDAC inhibitors (vorinostat, panobinostat)	Promote chromatin relaxation; reactivation of silenced genes	Investigational; limited clinical activity; no meaningful objective responses	[150,151,152]
	BRD9 degraders (FHD-609, CFT8634)	Disrupt BRD9-dependent SWI/SNF (BAF) chromatin remodeling and transcriptional programs	Early-phase trials; limited activity with QTc-related safety concerns; combinations under evaluation	[67]
Immunotherapy	ICIs (pembrolizumab, nivolumab, ipilimumab)	PD-1/PD-L1 and CTLA-4 blockade	Advanced or metastatic disease; limited clinical efficacy; associated with “cold” TME and low TMB	[153,154]
	NY-ESO-1–directed TCR therapy	HLA-restricted TCR targeting a cancer-testis antigen	Advanced or metastatic disease; promising clinical activity with durable responses	[155]
	Afamitresgene autoleucel (afami-cel)	Engineered TCR targeting MAGE-A4	Advanced or metastatic disease; durable responses in heavily pretreated MAGE-A4–positive SS; FDA accelerated approval (2024)	[156]
Other emerging targeted therapy	ATR inhibitors (berzosertib/VX970/VE-822)	ATR dependency driven by *SS18*::*SSX*-associated replication stress; induction of DNA damage (γH2AX) and apoptosis	Early-phase trials; tumor growth suppression in PDX models; synergy with cisplatin or PARP inhibitors	[159,160]
	CDK inhibitors (CDK4/6 [palbociclib], CDK7, CDK9)	Disrupt cell cycle control and transcriptional programs	Early-phase studies; preclinical: suppression of proliferation; preliminary clinical activity of palbociclib	[161,162,163,164,165,166]
	IGF-1R inhibitors (figitumumab and cixutumumab)	Anti–IGF-1R monoclonal antibodies; inhibit IGF-1R–mediated PI3K/AKT/mTOR and RAS/MAPK signaling	Investigational; limited efficacy in SS	[169,170]

TKI, tyrosine kinase inhibitor; VEGFR, vascular endothelial growth factor receptor; PDGFR, platelet-derived growth factor receptor; FGFR, fibroblast growth factor receptor; PFS, progression-free survival; KIT, KIT proto-oncogene receptor tyrosine kinase; RAF1, Raf-1 proto-oncogene, serine/threonine kinase; TIE2, tyrosine kinase with immunoglobulin-like and EGF-like domains 2; EZH2, enhancer of zeste homolog 2; HDAC, histone deacetylase; BRD9, bromodomain-containing 9; SWI/SNF, Switch/sucrose non-fermentable; BAF, BRG1- or BRM-associated factor; QTc, corrected QT interval; ICI, immune checkpoint inhibitor; PD-1, programmed cell death protein 1; PD-L1, programmed death-ligand 1; CTLA-4, cytotoxic T-lymphocyte–associated protein 4; TME, tumor microenvironment; TMB, tumor mutational burden; NY-ESO-1, New York esophageal squamous cell carcinoma-1; TCR, T cell receptor; HLA, human leukocyte antigen; MAGE-A4, melanoma-associated antigen A4; SS, synovial sarcoma; ATR, ataxia telangiectasia and Rad3-related protein; γH2AX, phosphorylated histone H2AX; PDX, patient-derived xenograft; PARP, poly(ADP-ribose) polymerase; IGF-1R, insulin-like growth factor-1R; PI3K, phosphoinositide 3-kinase; AKT, protein kinase B (PKB); mTOR, mechanistic target of rapamycin; RAS, RAS proto-oncogene, GTPase; MAPK, mitogen-activated protein kinase.

## Data Availability

No new data were created or analyzed in this study. Data sharing is not applicable to this article.

## Abbreviations

The following abbreviations are used in this manuscript:

AKT	Protein kinase B
ATF2	Activating transcription factor 2
ATR	Ataxia telangiectasia and Rad3-related protein
BAF	BRG1/BRM-associated factor complex (SWI/SNF complex)
BAP1	BRCA1-associated protein 1
BCL2	B-cell lymphoma 2
BCOR	BCL6 corepressor
BRAF	B-Raf proto-oncogene, serine/threonine kinase
BRD	Bromodomain
BRD9	Bromodomain-containing protein 9
CCNB3	Cyclin B3
CDK	Cyclin-dependent kinase
CK	Cytokeratin
CTLA-4	Cytotoxic T-lymphocyte-associated protein 4
DCE-MRI	Dynamic contrast-enhanced magnetic resonance imaging
CpG	Cytosine–phosphate–guanine
CT	Computed tomography
DFSP	Dermatofibrosarcoma protuberans
DOG1	Discovered on GIST-1
EBRT	External beam radiotherapy
EMA	Epithelial membrane antigen
ERG	ETS-related gene
ESMO	European Society for Medical Oncology
EURACAN	European Reference Network for Rare Adult Solid Cancers
EWSR1	Ewing sarcoma breakpoint region 1
EZH2	Enhancer of zeste homolog 2
FGFR	Fibroblast growth factor receptor
FNCLCC	Fédération Nationale des Centres de Lutte Contre le Cancer
GENTURIS	European Reference Network on Genetic Tumour Risk Syndromes
GI	Gastrointestinal
H3K27me3	Trimethylation of histone H3 at lysine 27
H&E	Hematoxylin and eosin
HDAC	Histone deacetylase
HLA	Human leukocyte antigen
HPF	High-power field
γH2AX	Phosphorylated histone H2AX
ICI	Immune checkpoint inhibitor
IHC	Immunohistochemistry
INI1	Integrase interactor 1
KDM2B	Lysine demethylase 2B
KIT	KIT proto-oncogene receptor tyrosine kinase
MAGEA1	Melanoma antigen gene A1
MAGEA4	Melanoma antigen gene A4
MAPK	Mitogen-activated protein kinase
MN1	Meningioma 1
MPNST	Malignant peripheral nerve sheath tumor
MRI	Magnetic resonance imaging
mTOR	Mechanistic target of rapamycin
ncBAF	Non-canonical BRG1/BRM-associated factor complex
NEDD4	Neural precursor cell expressed developmentally downregulated protein 4
NTRK	Neurotrophic tyrosine receptor kinase
NY-ESO-1	New York–esophageal squamous cell carcinoma-1
PARP	Poly(ADP-ribose) polymerase
PD-1	Programmed cell death protein 1
PD-L1	Programmed death-ligand 1
PDX	Patient-derived xenograft
PERSARC	PERsonalized SARcoma Care
PFS	Progression-free survival
PI3K	Phosphoinositide 3-kinase
PRAME	Preferentially expressed antigen in melanoma
RAF1	Raf-1 proto-oncogene, serine/threonine kinase
SFT	Solitary fibrous tumor
SMA	Smooth muscle actin
SMARCB1	SWI/SNF-related, matrix-associated, actin-dependent regulator of chromatin, subfamily B, member 1
SARCULATOR	Sarcoma calculator
SATB2	Special AT-rich sequence-binding protein 2
SOX10	SRY-box transcription factor 10
SS	Synovial sarcoma
SS18	Synovial sarcoma translocation, chromosome 18
SSX	Synovial sarcoma, X breakpoint
SWI/SNF	SWItch/sucrose non-fermentable chromatin-remodeling complex
TCR	T cell receptor
TIE2	Tyrosine kinase with immunoglobulin-like and EGF-like domains 2
TKI	Tyrosine kinase inhibitor
TLE1	Transducin-like enhancer of split 1
TMB	Tumor mutational burden
TME	Tumor microenvironment
TRPS1	Trichorhinophalangeal syndrome type 1
VEGFR	Vascular endothelial growth factor receptor
WT1	Wilms tumor 1

## References

[1] Suurmeijer A.J.H., Ladanyi M., Nielsen T.O. (2020). Synovial sarcoma. WHO Classification of Tumours. Soft Tissue and Bone Tumours.

[2] Sultan I., Rodriguez-Galindo C., Saab R., Yasir S., Casanova M., Ferrari A. (2009). Comparing children and adults with synovial sarcoma in the Surveillance, Epidemiology, and End Results program, 1983 to 2005: An analysis of 1268 patients. Cancer.

[3] Thway K., Fisher C. (2014). Synovial sarcoma: Defining features and diagnostic evolution. Ann. Diagn. Pathol..

[4] Ghadially F.N., Roy S. (1966). Experimentally produced synovial sarcomas. Cancer.

[5] Leader M., Patel J., Collins M., Kristin H. (1987). Synovial sarcomas. True carcinosarcomas?. Cancer.

[6] Smith M.E., Fisher C., Wilkinson L.S., Edwards J.C. (1995). Synovial sarcomas lack synovial differentiation. Histopathology.

[7] Hajdu S.I., Shiu M.H., Fortner J.G. (1977). Tendosynovial sarcoma: A clinicopathological study of 136 cases. Cancer.

[8] Dische F.E., Darby A.J., Howard E.R. (1978). Malignant synovioma: Electron microscopical findings in three patients and review of the literature. J. Pathol..

[9] Miettinen M., Fetsch J.F., Antonescu C.R., Folpe A.L., Wakely P.E. (2014). Tumors of the Soft Tissues.

[10] Simon G. (1865). Exstirpation einer sehr grossen, mit dicken Stiele angewachsenen Kniegelenkmaus mit glücklichem Erfolge. Arch. Klin. Chir..

[11] Lejars F., Rubens-Duval H. (1910). Les sarcomes primitifs des synoviales articulaires. Rev. Chir..

[12] Smith L.W. (1927). Synoviomata. Am. J. Pathol..

[13] Sabrazes J., Loubat E., de Grailly R., Magendie J. (1934). Synovial sarcomes. Gaz. Hebd. Sci. Méd. Bordx..

[14] Knox L.C. (1936). Synovial sarcoma. Am. J. Cancer.

[15] Haagensen C.D., Stout A.P. (1944). Synovial Sarcoma. Ann. Surg..

[16] King E.S. (1952). Tissue differentiation in malignant synovial tumours. J. Bone J0it. Surg..

[17] Cade S. (1962). Synovial sarcoma. J. R. Coll. Surg. Edinb..

[18] Mackenzie D.H. (1966). Synovial sarcoma. A review of 58 cases. Cancer.

[19] Evans H.L. (1980). Synovial sarcoma. A study of 23 biphasic and 17 probable monophasic examples. Pathol. Annu..

[20] Krall R.A., Kostianovsky M., Patchefsky A.S. (1981). Synovial sarcoma: A clinical, pathological, and ultrastructural study of 26 cases supporting the recognition of a monophasic variant. Am. J. Surg. Pathol..

[21] Cadman N.L., Soule E.H., Kelly P.J. (1965). Synovial sarcoma; an analysis of 34 tumors. Cancer.

[22] Schmidt D., Mackay B. (1982). Ultrastructure of human tendon sheath and synovium: Implications for tumor histogenesis. Ultrastruct. Pathol..

[23] Kerouanton A., Jimenez I., Cellier C., Laurence V., Helfre S., Pannier S., Mary P., Freneaux P., Orbach D. (2014). Synovial sarcoma in children and adolescents. J. Pediatr. Hematol. Oncol..

[24] Zhang G., Xiao B., Huang H., Zhang Y., Zhang X., Zhang J., Wang Y. (2019). Intracranial synovial sarcoma: A clinical, radiological and pathological study of 16 cases. Eur. J. Surg. Oncol..

[25] Fisher C., Folpe A.L., Hashimoto H., Weiss S.W. (2004). Intra-abdominal synovial sarcoma: A clinicopathological study. Histopathology.

[26] Bégueret H., Galateau-Salle F., Guillou L., Chetaille B., Brambilla E., Vignaud J.M., Terrier P., Groussard O., Coindre J.M. (2005). Primary intrathoracic synovial sarcoma: A clinicopathologic study of 40 t(X;18)-positive cases from the French Sarcoma Group and the Mesopath Group. Am. J. Surg. Pathol..

[27] Chrisinger J.S.A., Salem U.I., Kindblom L.G., Amini B., Hansson M., Meis J.M. (2017). Synovial Sarcoma of Peripheral Nerves: Analysis of 15 Cases. Am. J. Surg. Pathol..

[28] Righi A., Gambarotti M., Benini S., Gibertoni D., Asioli S., Magagnoli G., Gamberi G., Sbaraglia M., Cocchi S., Staals E. (2022). Primary synovial sarcoma of bone: A retrospective analysis of 25 patients. Histopathology.

[29] Sharma A., Ko J.S., Billings S.D. (2021). Primary cutaneous synovial sarcoma-Sometimes the hoof beats are zebras. J. Cutan. Pathol..

[30] Pors J., Devereaux K.A., Hildebrandt D., Longacre T.A. (2022). Primary uterine synovial sarcoma with SMARCA4 loss. Histopathology.

[31] Kolin D.L., Crum C.P., Baranov E., Cin P.D., Chang M.C., Colgan T.J., Dickson B.C., Hornick J.L., Nucci M.R. (2020). Synovial sarcoma of the female genital tract: A protean mimic of Müllerian neoplasia. Am. J. Surg. Pathol..

[32] Miettinen M., Sciot R., Tsui W.M. (2019). Synovial sarcoma. WHO Classification of Tumours. Digestive System Tumours.

[33] Wang J.G., Li N.N. (2013). Primary cardiac synovial sarcoma. Ann. Thorac. Surg..

[34] Lan T., Chen H., Xiong B., Zhou T., Peng R., Chen M., Ye F., Yao J., He X., Wang Y. (2016). Primary pleuropulmonary and mediastinal synovial sarcoma: A clinicopathologic and molecular study of 26 genetically confirmed cases in the largest institution of southwest China. Diagn. Pathol..

[35] Terra S., Aesif S.W., Maleszewski J.J., Folpe A.L., Boland J.M. (2018). Mediastinal synovial sarcoma: Clinicopathologic analysis of 21 cases with molecular confirmation. Am. J. Surg. Pathol..

[36] Hartel P.H., Fanburg-Smith J.C., Frazier A.A., Galvin J.R., Lichy J.H., Shilo K., Franks T.J. (2007). Primary pulmonary and mediastinal synovial sarcoma: A clinicopathologic study of 60 cases and comparison with five prior series. Mod. Pathol..

[37] Argani P., Faria P.A., Epstein J.I., Reuter V.E., Perlman E.J., Beckwith J.B., Ladanyi M. (2000). Primary renal synovial sarcoma: Molecular and morphologic delineation of an entity previously included among embryonal sarcomas of the kidney. Am. J. Surg. Pathol..

[38] Iacovelli R., Altavilla A., Ciardi A., Urbano F., Manai C., Gentile V., Cortesi E. (2012). Clinical and pathological features of primary renal synovial sarcoma: Analysis of 64 cases from 11 years of medical literature. BJU Int..

[39] Schoolmeester J.K., Cheville J.C., Folpe A.L. (2014). Synovial sarcoma of the kidney: A clinicopathologic, immunohistochemical, and molecular genetic study of 16 cases. Am. J. Surg. Pathol..

[40] Rose L., Grignon D., Cheng L., Fan R., Zhang S., Alruwaii F., Chen S. (2020). Primary renal synovial sarcomas: PAX 8 immunostaining and unusual molecular findings. Appl. Immunohistochem. Mol. Morphol..

[41] Olofson A.M., Linos K. (2017). Primary Intraprostatic Synovial Sarcoma. Arch. Pathol. Lab. Med..

[42] Salcedo-Hernández R.A., Lino-Silva L.S., Luna-Ortiz K. (2013). Synovial sarcomas of the head and neck: Comparative analysis with synovial sarcoma of the extremities. Auris Nasus Larynx.

[43] Gopalakrishnan V., Amini B., Wagner M.J., Nowell E.N., Lazar A.J., Lin P.P., Benjamin R.S., Araujo D.M. (2017). Synovial sarcoma of the head and neck: A single institution review. Sarcoma.

[44] Patel R.R., Gopalakrishnan V., Amini B., Lazar A.J., Lin P.P., Benjamin R.S., Bishop A.J., Goepfert R.P., Araujo D.M. (2024). Oncologic outcomes in patients with localized, primary head and neck synovial sarcoma. Cancers.

[45] Gervasio K.A., Ramesh S., Sivalingam M.D., Markovitz M., Milman T. (2021). Primary synovial sarcoma of the orbit: A case report and update on diagnostic techniques. Ophthalmic Plast. Reconstr. Surg..

[46] Stagner A.M., Jakobiec F.A., Fay A. (2017). Primary orbital synovial sarcoma: A clinicopathologic review with a differential diagnosis and discussion of molecular genetics. Surv. Ophthalmol..

[47] Wladis E.J., Farber M.G., Nepo A.G. (2012). Metastatic synovial sarcoma to the orbit. Ophthalmic Plast. Reconstr. Surg..

[48] Bixby S.D., Hettmer S., Taylor G.A., Voss S.D. (2010). Synovial sarcoma in children: Imaging features and common benign mimics. Am. J. Roentgenol..

[49] Yoshida A., Klebe S., Ladanyi M., Suurmeijer A.J.H. (2021). Synovial sarcoma of the thorax. WHO Classification of Tumours. Thoracic Tumours.

[50] Bullerdiek J., Bell D. (2023). Synovial sarcoma. WHO Classification of Tumours. Head and Neck Tumours.

[51] Wilkerson B.W., Crim J.R., Hung M., Layfield L.J. (2012). Characterization of synovial sarcoma calcification. Am. J. Roentgenol..

[52] Hisaoka M., Matsuyama A., Shimajiri S., Akiba J., Kusano H., Hiraoka K., Shoda T., Hashimoto H. (2009). Ossifying synovial sarcoma. Pathol. Res. Pract..

[53] Kind M., Stock N., Coindre J.M. (2009). Histology and imaging of soft tissue sarcomas. Eur. J. Radiol..

[54] Liang C., Mao H., Tan J., Ji Y., Sun F., Dou W., Wang H., Wang H., Gao J. (2015). Synovial sarcoma: Magnetic resonance and computed tomography imaging features and differential diagnostic considerations. Oncol. Lett..

[55] Murphey M.D., Gibson M.S., Jennings B.T., Crespo-Rodríguez A.M., Fanburg-Smith J., Gajewski D.A. (2006). From the archives of the AFIP: Imaging of synovial sarcoma with radiologic-pathologic correlation. Radiographics.

[56] Herrera-Goepfert R. (2018). Postradiation synovial sarcoma of the common bile duct: A previously unreported anatomic site. Int. J. Surg. Pathol..

[57] Ladanyi M., Antonescu C.R., Leung D.H., Woodruff J.M., Kawai A., Healey J.H., Brennan M.F., Bridge J.A., Neff J.R., Barr F.G. (2002). Impact of *SYT-SSX* fusion type on the clinical behavior of synovial sarcoma: A multi-institutional retrospective study of 243 patients. Cancer Res..

[58] dos Santos N.R., de Bruijn D.R., van Kessel A.G. (2001). Molecular mechanisms underlying human synovial sarcoma development. Genes Chromosomes Cancer.

[59] Amary M.F., Berisha F., Bernardi F.D.C., Herbert A., James M., Reis-Filho J.S., Fisher C., Nicholson A.G., Tirabosco R., Diss T.C. (2007). Detection of *SS18-SSX* fusion transcripts in formalin-fixed paraffin-embedded neoplasms: Analysis of conventional RT-PCR, qRT-PCR and dual color FISH as diagnostic tools for synovial sarcoma. Mod. Pathol..

[60] The Cancer Genome Atlas Research Network (2017). Comprehensive and integrated genomic characterization of adult soft tissue sarcomas. Cell.

[61] Waterfall J.J., Meltzer P.S. (2012). Targeting epigenetic misregulation in synovial sarcoma. Cancer Cell.

[62] Jones K.B., Barrott J.J., Xie M., Haldar M., Jin H., Zhu J.F., Monument M.J., Mosbruger T.L., Langer E.M., Randall R.L. (2016). The impact of chromosomal translocation locus and fusion oncogene coding sequence in synovial sarcomagenesis. Oncogene.

[63] Barrott J.J., Illum B.E., Jin H., Hedberg M.L., Wang Y., Grossmann A., Haldar M., Capecchi M.R., Jones K.B. (2018). Paracrine osteoprotegerin and β-catenin stabilization support synovial sarcomagenesis in periosteal cells. J. Clin. Investig..

[64] Nagai M., Tanaka S., Tsuda M., Endo S., Kato H., Sonobe H., Minami A., Hiraga H., Nishihara H., Sawa H. (2001). Analysis of transforming activity of human synovial sarcoma-associated chimeric protein SYT-SSX1 bound to chromatin remodeling factor hBRM/hSNF2α. Proc. Natl. Acad. Sci. USA.

[65] Kadoch C., Crabtree G.R. (2013). Reversible disruption of mSWI/SNF (BAF) complexes by the *SS18-SSX* oncogenic fusion in synovial sarcoma. Cell.

[66] Michel B.C., D’Avino A.R., Cassel S.H., Mashtalir N., McKenzie Z.M., McBride M.J., Valencia A.M., Zhou Q., Bocker M., Soares L.M.M. (2018). A non-canonical SWI/SNF complex is a synthetic lethal target in cancers driven by BAF complex perturbation. Nat. Cell Biol..

[67] Brien G.L., Remillard D., Shi J., Hemming M.L., Chabon J., Wynne K., Dillon E.T., Cagney G., Van Mierlo G., Baltissen M.P. (2018). Targeted degradation of BRD9 reverses oncogenic gene expression in synovial sarcoma. Elife.

[68] Nielsen T.O., Poulin N.M., Ladanyi M. (2015). Synovial sarcoma: Recent discoveries as a roadmap to new avenues for therapy. Cancer Discov..

[69] Banito A., Li X., Laporte A.N., Roe J.S., Sanchez-Vega F., Huang C.H., Dancsok A.R., Hatzi K., Chen C.C., Tschaharganeh D.F. (2018). The SS18-SSX Oncoprotein hijacks KDM2B-PRC1.1 to drive synovial sarcoma. Cancer Cell.

[70] McBride M.J., Pulice J.L., Beird H.C., Ingram D.R., D’Avino A.R., Shern J.F., Charville G.W., Hornick J.L., Nakayama R.T., Garcia-Rivera E.M. (2018). The SS18-SSX fusion oncoprotein hijacks BAF complex targeting and function to drive synovial sarcoma. Cancer Cell.

[71] Storlazzi C.T., Mertens F., Mandahl N., Gisselsson D., Isaksson M., Gustafson P., Domanski H.A., Panagopoulos I. (2003). A novel fusion gene, *SS18L1/SSX1*, in synovial sarcoma. Genes Chromosomes Cancer.

[72] Antonescu C.R., Kawai A., Leung D.H., Lonardo F., Woodruff J.M., Healey J.H., Ladanyi M. (2000). Strong association of *SYT-SSX* fusion type and morphologic epithelial differentiation in synovial sarcoma. Diagn. Mol. Pathol..

[73] Yoshida A., Arai Y., Satomi K., Kubo T., Ryo E., Matsushita Y., Hama N., Sudo K., Komiyama M., Yatabe Y. (2022). Identification of novel *SSX1* fusions in synovial sarcoma. Mod. Pathol..

[74] Argani P., Zhang L., Sung Y.S., Bacchi C., Swanson D., Dickson B.C., Antonescu C.R. (2020). Novel SS18-NEDD4 gene fusion in a primary renal synovial sarcoma. Genes Chromosomes Cancer.

[75] Lagarde P., Przybyl J., Brulard C., Pérot G., Pierron G., Delattre O., Sciot R., Wozniak A., Schöffski P., Terrier P. (2013). Chromosome instability accounts for reverse metastatic outcomes of pediatric and adult synovial sarcomas. J. Clin. Oncol..

[76] Orbach D., Mosseri V., Pissaloux D., Pierron G., Brennan B., Ferrari A., Chibon F., Bisogno G., De Salvo G.L., Chakiba C. (2018). Genomic complexity in pediatric synovial sarcomas (Synobio study): The European pediatric soft tissue sarcoma group (EpSSG) experience. Cancer Med..

[77] Watanabe S., Shimomura A., Kubo T., Sekimizu M., Seo T., Watanabe S.I., Kawai A., Yamamoto N., Tamura K., Kohno T. (2020). BRAF V600E mutation is a potential therapeutic target for a small subset of synovial sarcoma. Mod. Pathol..

[78] Michal M., Fanburg-Smith J.C., Lasota J., Fetsch J.F., Lichy J., Miettinen M. (2006). Minute synovial sarcomas of the hands and feet: A clinicopathologic study of 21 tumors less than 1 cm. Am. J. Surg. Pathol..

[79] Majeste R.M., Beckman E.N. (1988). Synovial sarcoma with an overwhelming epithelial component. Cancer.

[80] Weidner N., Goldman R., Johnston J. (1993). Epithelioid monophasic synovial sarcoma. Ultrastruct. Pathol..

[81] Chow L.T. (2015). Primary synovial epithelioid sarcoma of the knee: Distinctly unusual location leading to its confusion with pigmented villonodular synovitis. APMIS.

[82] Krane J.F., Bertoni F., Fletcher C.D. (1999). Myxoid synovial sarcoma: An underappreciated morphologic subset. Mod. Pathol..

[83] Milchgrub S., Ghandur-Mnaymneh L., Dorfman H.D., Albores-Saavedra J. (1993). Synovial sarcoma with extensive osteoid and bone formation. Am. J. Surg. Pathol..

[84] Qassid O., Ali A., Thway K. (2016). Synovial Sarcoma with Myoid Differentiation. Int. J. Surg. Pathol..

[85] Chen Y., Zhou N., Guo D., Wang X., He X., Xu Y. (2022). Predominantly epithelial-type synovial sarcoma with overwhelming neuroendocrine differentiation: A potential diagnostic pitfall. Diagn. Pathol..

[86] Satoh H., Takayashiki N., Shiozawa T., Miyazaki K., Ohara G., Kagohashi K., Kurishima K., Sugita S., Aoyama T., Hasegawa T. (2015). Recurrent pulmonary synovial sarcoma effectively treated with amrubicin: A case report. Exp. Ther. Med..

[87] Mirra J.M., Wang S., Bhuta S. (1984). Synovial sarcoma with squamous differentiation of its mesenchymal glandular elements. A case report with light-microscopic, ultramicroscopic, and immunologic correlation. Am. J. Surg. Pathol..

[88] Guillou L., Benhattar J., Bonichon F., Gallagher G., Terrier P., Stauffer E., Somerhausen Nde S., Michels J.J., Jundt G., Vince D.R. (2004). Histologic grade, but not SYT-SSX fusion type, is an important prognostic factor in patients with synovial sarcoma: A multicenter, retrospective analysis. J. Clin. Oncol..

[89] Bergh P., Meis-Kindblom J.M., Gherlinzoni F., Berlin O., Bacchini P., Bertoni F., Gunterberg B., Kindblom L.G. (1999). Synovial sarcoma: Identification of low and high risk groups. Cancer.

[90] de Silva M.V., McMahon A.D., Paterson L., Reid R. (2003). Identification of poorly differentiated synovial sarcoma: A comparison of clinicopathological and cytogenetic features with those of typical synovial sarcoma. Histopathology.

[91] Chan J.A., McMenamin M.E., Fletcher C.D. (2003). Synovial sarcoma in older patients: Clinicopathological analysis of 32 cases with emphasis on unusual histological features. Histopathology.

[92] Choi J.H., Shim Y.R., Bae Y.K., Kim M.J., Shin D.S., Cho K.H. (2005). Synovial sarcoma with massive myxoid feature: A case report. J. Pathol. Transl. Med..

[93] Coli A., Bigotti G., Parente R., Massi G. (2006). Myxoid monophasic synovial sarcoma: Case report of an unusual histological variant. J. Exp. Clin. Cancer Res..

[94] Varela-Duran J., Enzinger F.M. (1982). Calcifying synovial sarcoma. Cancer.

[95] Miettinen M., Limon J., Niezabitowski A., Lasota J. (2000). Patterns of keratin polypeptides in 110 biphasic, monophasic, and poorly differentiated synovial sarcomas. Virchows Arch..

[96] Pelmus M., Guillou L., Hostein I., Sierankowski G., Lussan C., Coindre J.M. (2002). Monophasic fibrous and poorly differentiated synovial sarcoma: Immunohistochemical reassessment of 60 t(X;18)(SYT-SSX)-positive cases. Am. J. Surg. Pathol..

[97] Terry J., Saito T., Subramanian S., Ruttan C., Antonescu C.R., Goldblum J.R., Downs-Kelly E., Corless C.L., Rubin B.P., van de Rijn M. (2007). TLE1 as a diagnostic immunohistochemical marker for synovial sarcoma emerging from gene expression profiling studies. Am. J. Surg. Pathol..

[98] Knösel T., Heretsch S., Altendorf-Hofmann A., Richter P., Katenkamp K., Katenkamp D., Berndt A., Petersen I. (2010). TLE1 is a robust diagnostic biomarker for synovial sarcomas and correlates with t(X;18): Analysis of 319 cases. Eur. J. Cancer.

[99] Matsuyama A., Hisaoka M., Iwasaki M., Iwashita M., Hisanaga S., Hashimoto H. (2010). TLE1 expression in malignant mesothelioma. Virchows Arch..

[100] Kosemehmetoglu K., Vrana J.A., Folpe A.L. (2009). TLE1 expression is not specific for synovial sarcoma: A whole section study of 163 soft tissue and bone neoplasms. Mod. Pathol..

[101] Klebe S., Prabhakaran S., Hocking A., Pulford E., Moore S., Nicola M., Allen P.W., Henderson D.W. (2018). Pleural malignant mesothelioma versus pleuropulmonary synovial sarcoma: A clinicopathological study of 22 cases with molecular analysis and survival data. Pathology.

[102] Foo W.C., Cruise M.W., Wick M.R., Hornick J.L. (2011). Immunohistochemical staining for TLE1 distinguishes synovial sarcoma from histologic mimics. Am. J. Clin. Pathol..

[103] Baranov E., McBride M.J., Bellizzi A.M., Ligon A.H., Fletcher C.D.M., Kadoch C., Hornick J.L. (2020). A Novel SS18-SSX Fusion-specific Antibody for the Diagnosis of Synovial Sarcoma. Am. J. Surg. Pathol..

[104] Maclean F., Chou A., Gill A.J. (2020). When used together SS18-SSX fusion-specific and SSX C-terminus immunohistochemistry are highly specific and sensitive for the diagnosis of synovial sarcoma and can replace FISH or molecular testing in most cases. Histopathology.

[105] Lasota J., Chłopek M., Kaczorowski M., Natálie K., Ryś J., Kopczyński J., Sulaieva O., Michal M., Kruczak A., Harazin-Lechowska A. (2024). Utility of immunohistochemistry with antibodies to SS18-SSX chimeric proteins and C-terminus of SSX protein for synovial sarcoma differential diagnosis. Am. J. Surg. Pathol..

[106] Miettinen M., Limon J., Niezabitowski A., Lasota J. (2001). Calretinin and other mesothelioma markers in synovial sarcoma: Analysis of antigenic similarities and differences with malignant mesothelioma. Am. J. Surg. Pathol..

[107] Olsen S.H., Thomas D.G., Lucas D.R. (2006). Cluster analysis of immunohistochemical profiles in synovial sarcoma, malignant peripheral nerve sheath tumor, and Ewing sarcoma. Mod. Pathol..

[108] Kohashi K., Oda Y., Yamamoto H., Tamiya S., Matono H., Iwamoto Y., Taguchi T., Tsuneyoshi M. (2010). Reduced expression of SMARCB1/INI1 protein in synovial sarcoma. Mod. Pathol..

[109] Arnold M.A., Arnold C.A., Li G., Chae U., El-Etriby R., Lee C.C., Tsokos M. (2013). A unique pattern of INI1 immunohistochemistry distinguishes synovial sarcoma from its histologic mimics. Hum. Pathol..

[110] Mustapar N., Zawawi M.S.F., Tuan Sharif S.E. (2020). The value of H3K27me3 immunohistochemistry in differentiating malignant peripheral nerve sheath tumour with its histologic mimickers. Asian Pac. J. Cancer Prev..

[111] Ito J., Asano N., Kawai A., Yoshida A. (2016). The diagnostic utility of reduced immunohistochemical expression of SMARCB1 in synovial sarcomas: A validation study. Hum. Pathol..

[112] Ai D., Yao J., Yang F., Huo L., Chen H., Lu W., Soto L.M.S., Jiang M., Raso M.G., Wang S. (2021). TRPS1: A highly sensitive and specific marker for breast carcinoma, especially for triple-negative breast cancer. Mod. Pathol..

[113] Cloutier J.M., Ingram D.R., Wani K., Lazar A.J., Wang W.L. (2022). Frequent TRPS1 expression in synovial sarcoma is associated with *SS18-SSX* fusion oncoprotein activity. Hum. Pathol..

[114] Iura K., Maekawa A., Kohashi K., Ishii T., Bekki H., Otsuka H., Yamada Y., Yamamoto H., Harimaya K., Iwamoto Y. (2017). Cancer-testis antigen expression in synovial sarcoma: NY-ESO-1, PRAME, MAGEA4, and MAGEA1. Hum. Pathol..

[115] van de Rijn M., Barr F.G., Xiong Q.B., Hedges M., Shipley J., Fisher C. (1999). Poorly differentiated synovial sarcoma: An analysis of clinical, pathologic, and molecular genetic features. Am. J. Surg. Pathol..

[116] Italiano A., Di Mauro I., Rapp J., Pierron G., Auger N., Alberti L., Chibon F., Escande F., Voegeli A.C., Ghnassia J.P. (2016). Clinical effect of molecular methods in sarcoma diagnosis (GENSARC): A prospective, multicentre, observational study. Lancet Oncol..

[117] Fiore M., Sambri A., Spinnato P., Zucchini R., Giannini C., Caldari E., Pirini M.G., De Paolis M. (2021). The biology of synovial sarcoma: State-of-the-art and future perspectives. Curr. Treat. Options Oncol..

[118] Blay J.Y., von Mehren M., Jones R.L., Martin-Broto J., Stacchiotti S., Bauer S., Gelderblom H., Orbach D., Hindi N., Dei Tos A. (2023). Synovial sarcoma: Characteristics, challenges, and evolving therapeutic strategies. ESMO Open.

[119] Fuchs J.R., Schulte B.C., Fuchs J.W., Agulnik M. (2023). Emerging targeted and cellular therapies in the treatment of advanced and metastatic synovial sarcoma. Front. Oncol..

[120] Landuzzi L., Manara M.C., Pazzaglia L., Lollini P.L., Scotlandi K. (2023). Innovative breakthroughs for the treatment of advanced and metastatic synovial sarcoma. Cancers.

[121] Ren C., Liu J., Hornicek F.J., Yue B., Duan Z. (2024). Advances of *SS18-SSX* fusion gene in synovial sarcoma: Emerging novel functions and therapeutic potentials. Biochim. Biophys. Acta Rev. Cancer.

[122] Pretell-Mazzini J., Barton M.D., Conway S.A., Temple H.T. (2015). Unplanned excision of soft-tissue sarcomas: Current concepts for management and prognosis. J. Bone Jt. Surg. Am..

[123] Von Mehren M., Randall R.L., Benjamin R.S., Boles S., Bui M.M., Conrad E.U., Ganjoo K.N., George S., Gonzalez R.J., Heslin M.J. (2016). Soft tissue sarcoma, version 2.2016, NCCN clinical practice guidelines in oncology. J. Natl. Compr. Cancer Netw..

[124] Ferrari A., Chi Y.Y., De Salvo G.L., Orbach D., Brennan B., Randall R.L., McCarville M.B., Black J.O., Alaggio R., Hawkins D.S. (2017). Surgery alone is sufficient therapy for children and adolescents with low-risk synovial sarcoma: A joint analysis from the European paediatric soft tissue sarcoma Study Group and the Children’s Oncology Group. Eur. J. Cancer.

[125] Kawaguchi N., Ahmed A.R., Matsumoto S., Manabe J., Matsushita Y. (2004). The concept of curative margin in surgery for bone and soft tissue sarcoma. Clin. Orthop. Relat. Res..

[126] O’Donnell P.W., Griffin A.M., Eward W.C., Sternheim A., Catton C.N., Chung P.W., O’Sullivan B., Ferguson P.C., Wunder J.S. (2014). The effect of the setting of a positive surgical margin in soft tissue sarcoma. Cancer.

[127] Gingrich A.A., Marrufo A.S., Liu Y., Li C.S., Darrow M.A., Monjazeb A.M., Thorpe S.W., Canter R.J. (2020). Radiotherapy is associated with improved survival in patients with synovial sarcoma undergoing surgery: A national cancer database analysis. J. Surg. Res..

[128] Davis A.M., O’Sullivan B., Turcotte R., Bell R., Catton C., Chabot P., Wunder J., Hammond A., Benk V., Kandel R. (2005). Late radiation morbidity following randomization to preoperative versus postoperative radiotherapy in extremity soft tissue sarcoma. Radiother. Oncol..

[129] Wang J., Song Y., Liu X., Jin J., Wang W., Yu Z., Liu Y., Li N., Fang H., Ren H. (2019). Comparison of outcome and toxicity of postoperative intensity-modulated radiation therapy with two-dimensional radiotherapy in patients with soft tissue sarcoma of extremities and trunk. Cancer Med..

[130] O’Sullivan B., Griffin A.M., Dickie C.I., Sharpe M.B., Chung P.W., Catton C.N., Ferguson P.C., Wunder J.S., Deheshi B.M., White L.M. (2013). Phase 2 study of preoperative image-guided intensity-modulated radiation therapy to reduce wound and combined modality morbidities in lower extremity soft tissue sarcoma. Cancer.

[131] Stacchiotti S., Van Tine B.A. (2018). Synovial sarcoma: Current concepts and future perspectives. J. Clin. Oncol..

[132] Casali P.G., Abecassis N., Aro H.T., Bauer S., Biagini R., Bielack S., Bonvalot S., Boukovinas I., Bovee J., Brodowicz T. (2018). Soft tissue and visceral sarcomas: ESMO-EURACAN clinical practice guidelines for diagnosis, treatment and follow-up. Ann. Oncol..

[133] Edmonson J.H., Ryan L.M., Falkson C.I., Hicks D.G., Blum R.H. (2003). Phase II Study of ifosfamide + doxorubicin in patients with advanced synovial sarcomas (E1793): A trial of the Eastern Cooperative Oncology Group. Sarcoma.

[134] Sleijfer S., Ouali M., van Glabbeke M., Krarup-Hansen A., Rodenhuis S., Le Cesne A., Hogendoorn P.C., Verweij J., Blay J.Y. (2010). Prognostic and predictive factors for outcome to first-line ifosfamide-containing chemotherapy for adult patients with advanced soft tissue sarcomas: An exploratory, retrospective analysis on large series from the European Organization for Research and Treatment of Cancer-Soft Tissue and Bone Sarcoma Group (EORTC-STBSG). Eur. J. Cancer.

[135] Vlenterie M., Litière S., Rizzo E., Marréaud S., Judson I., Gelderblom H., Le Cesne A., Wardelmann E., Messiou C., Gronchi A. (2016). Outcome of chemotherapy in advanced synovial sarcoma patients: Review of 15 clinical trials from the European Organisation for Research and Treatment of Cancer Soft Tissue and Bone Sarcoma Group; setting a new landmark for studies in this entity. Eur. J. Cancer.

[136] Ferrari A., De Salvo G.L., Brennan B., van Noesel M.M., De Paoli A., Casanova M., Francotte N., Kelsey A., Alaggio R., Oberlin O. (2015). Synovial sarcoma in children and adolescents: The European Pediatric Soft Tissue Sarcoma Study Group prospective trial (EpSSG NRSTS 2005). Ann. Oncol..

[137] Gronchi A., Miah A.B., Dei Tos A.P., Abecassis N., Bajpai J., Bauer S., Biagini R., Bielack S., Blay J.Y., Bolle S. (2021). Soft tissue and visceral sarcomas: ESMO-EURACAN-GENTURIS Clinical Practice Guidelines for diagnosis, treatment and follow-up^☆^. Ann. Oncol..

[138] Maki R.G., Wathen J.K., Patel S.R., Priebat D.A., Okuno S.H., Samuels B., Fanucchi M., Harmon D.C., Schuetze S.M., Reinke D. (2007). Randomized phase II study of gemcitabine and docetaxel compared with gemcitabine alone in patients with metastatic soft tissue sarcomas: Results of sarcoma alliance for research through collaboration study 002. J. Clin. Oncol..

[139] Pender A., Davis E.J., Chauhan D., Messiou C., Al-Muderis O., Thway K., Fisher C., Zaidi S., Miah A., Judson I. (2018). Poor treatment outcomes with palliative gemcitabine and docetaxel chemotherapy in advanced and metastatic synovial sarcoma. Med. Oncol..

[140] Palmerini E., Sanfilippo R., Grignani G., Buonadonna A., Romanini A., Badalamenti G., Ferraresi V., Vincenzi B., Comandone A., Pizzolorusso A. (2021). Trabectedin for Patients with Advanced Soft Tissue Sarcoma: A Non-Interventional, Retrospective, Multicenter Study of the Italian Sarcoma Group. Cancers.

[141] Sanfilippo R., Dileo P., Blay J.Y., Constantinidou A., Le Cesne A., Benson C., Vizzini L., Contu M., Baldi G.G., Dei Tos A.P. (2015). Trabectedin in advanced synovial sarcomas: A multicenter retrospective study from four European institutions and the Italian Rare Cancer Network. Anticancer Drugs.

[142] van der Graaf W.T., Blay J.Y., Chawla S.P., Kim D.W., Bui-Nguyen B., Casali P.G., Schöffski P., Aglietta M., Staddon A.P., Beppu Y. (2012). Pazopanib for metastatic soft-tissue sarcoma (PALETTE): A randomised, double-blind, placebo-controlled phase 3 trial. Lancet.

[143] Gelderblom H., Judson I.R., Benson C., Merimsky O., Grignani G., Katz D., Freivogel K.W., Stein D., Jobanputra M., Mungul A. (2017). Treatment patterns and clinical outcomes with pazopanib in patients with advanced soft tissue sarcomas in a compassionate use setting: Results of the SPIRE study. Acta Oncol..

[144] Kasper B., Sleijfer S., Litière S., Marreaud S., Verweij J., Hodge R.A., Bauer S., Kerst J.M., van der Graaf W.T.A. (2014). Long-term responders and survivors on pazopanib for advanced soft tissue sarcomas: Subanalysis of two European Organisation for Research and Treatment of Cancer (EORTC) clinical trials 62043 and 62072. Ann. Oncol..

[145] Nakamura T., Matsumine A., Kawai A., Araki N., Goto T., Yonemoto T., Sugiura H., Nishida Y., Hiraga H., Honoki K. (2016). The clinical outcome of pazopanib treatment in Japanese patients with relapsed soft tissue sarcoma: A Japanese Musculoskeletal Oncology Group (JMOG) study. Cancer.

[146] Mir O., Brodowicz T., Italiano A., Wallet J., Blay J.Y., Bertucci F., Chevreau C., Piperno-Neumann S., Bompas E., Salas S. (2016). Safety and efficacy of regorafenib in patients with advanced soft tissue sarcoma (REGOSARC): A randomised, double-blind, placebo-controlled, phase 2 trial. Lancet Oncol..

[147] Xie C., Wan X., Quan H., Zheng M., Fu L., Li Y., Lou L. (2018). Preclinical characterization of anlotinib, a highly potent and selective vascular endothelial growth factor receptor-2 inhibitor. Cancer Sci..

[148] Changchien Y.C., Tátrai P., Papp G., Sápi J., Fónyad L., Szendrői M., Pápai Z., Sápi Z. (2012). Poorly differentiated synovial sarcoma is associated with high expression of enhancer of zeste homologue 2 (EZH2). J. Transl. Med..

[149] Shen J.K., Cote G.M., Gao Y., Choy E., Mankin H.J., Hornicek F.J., Duan Z. (2016). Targeting EZH2-mediated methylation of H3K27 inhibits proliferation and migration of Synovial Sarcoma in vitro. Sci. Rep..

[150] Su L., Sampaio A.V., Jones K.B., Pacheco M., Goytain A., Lin S., Poulin N., Yi L., Rossi F.M., Kast J. (2012). Deconstruction of the *SS18-SSX* fusion oncoprotein complex: Insights into disease etiology and therapeutics. Cancer Cell.

[151] Schmitt T., Mayer-Steinacker R., Mayer F., Grünwald V., Schütte J., Hartmann J.T., Kasper B., Hüsing J., Hajda J., Ottawa G. (2016). Vorinostat in refractory soft tissue sarcomas—Results of a multi-centre phase II trial of the German Soft Tissue Sarcoma and Bone Tumour Working Group (AIO). Eur. J. Cancer.

[152] Cassier P.A., Lefranc A., Amela E.Y., Chevreau C., Bui B.N., Lecesne A., Ray-Coquard I., Chabaud S., Penel N., Berge Y. (2013). A phase II trial of panobinostat in patients with advanced pretreated soft tissue sarcoma. A study from the French Sarcoma Group. Br. J. Cancer.

[153] Tawbi H.A., Burgess M., Bolejack V., Van Tine B.A., Schuetze S.M., Hu J., D’Angelo S., Attia S., Riedel R.F., Priebat D.A. (2017). Pembrolizumab in advanced soft-tissue sarcoma and bone sarcoma (SARC028): A multicentre, two-cohort, single-arm, open-label, phase 2 trial. Lancet Oncol..

[154] D’Angelo S.P., Mahoney M.R., Van Tine B.A., Atkins J., Milhem M.M., Jahagirdar B.N., Antonescu C.R., Horvath E., Tap W.D., Schwartz G.K. (2018). Nivolumab with or without ipilimumab treatment for metastatic sarcoma (Alliance A091401): Two open-label, non-comparative, randomised, phase 2 trials. Lancet Oncol..

[155] Dallos M., Tap W.D., D’Angelo S.P. (2016). Current status of engineered T-cell therapy for synovial sarcoma. Immunotherapy.

[156] D’Angelo S.P., Araujo D.M., Abdul Razak A.R., Agulnik M., Attia S., Blay J.Y., Carrasco Garcia I., Charlson J.A., Choy E., Demetri G.D. (2024). Afamitresgene autoleucel for advanced synovial sarcoma and myxoid round cell liposarcoma (SPEARHEAD-1): An international, open-label, phase 2 trial. Lancet.

[157] Pan M., Zhou M., Xie L., Bui N., Ganjoo K. (2024). Recent advances in sarcoma therapy: New agents, strategies and predictive biomarkers. J. Hematol. Oncol..

[158] Hazini A., Fisher K., Seymour L. (2021). Deregulation of HLA-I in cancer and its central importance for immunotherapy. J. Immunother. Cancer.

[159] Campbell J., Ryan C.J., Brough R., Bajrami I., Pemberton H.N., Chong I.Y., Costa-Cabral S., Frankum J., Gulati A., Holme H. (2016). Large-scale profiling of kinase dependencies in cancer cell lines. Cell Rep..

[160] Jones S.E., Fleuren E.D.G., Frankum J., Konde A., Williamson C.T., Krastev D.B., Pemberton H.N., Campbell J., Gulati A., Elliott R. (2017). ATR is a therapeutic target in synovial sarcoma. Cancer Res..

[161] Whittaker S.R., Mallinger A., Workman P., Clarke P.A. (2017). Inhibitors of cyclin-dependent kinases as cancer therapeutics. Pharmacol. Ther..

[162] Liao Y., Feng Y., Shen J., Hornicek F.J., Duan Z. (2016). The roles and therapeutic potential of cyclin-dependent kinases (CDKs) in sarcoma. Cancer Metastasis Rev..

[163] Li X., Seebacher N.A., Garbutt C., Ma H., Gao P., Xiao T., Hornicek F.J., Duan Z. (2018). Inhibition of cyclin-dependent kinase 4 as a potential therapeutic strategy for treatment of synovial sarcoma. Cell Death Dis..

[164] Li X., Dean D.C., Yuan J., Temple T.H., Trent J.C., Rosenberg A.E., Yu S., Hornicek F.J., Duan Z. (2022). Inhibition of CDK7-dependent transcriptional addiction is a potential therapeutic target in synovial sarcoma. Biomed. Pharmacother..

[165] Li X., Seebacher N.A., Xiao T., Hornicek F.J., Duan Z. (2019). Targeting regulation of cyclin dependent kinase 9 as a novel therapeutic strategy in synovial sarcoma. J. Orthop. Res..

[166] Vlenterie M., Hillebrandt-Roeffen M.H., Schaars E.W., Flucke U.E., Fleuren E.D., Navis A.C., Leenders W.P., Versleijen-Jonkers Y.M., van der Graaf W.T. (2016). Targeting cyclin-dependent kinases in synovial sarcoma: Palbociclib as a potential treatment for synovial sarcoma patients. Ann. Surg. Oncol..

[167] Friedrichs N., Küchler J., Endl E., Koch A., Czerwitzki J., Wurst P., Metzger D., Schulte J.H., Holst M.I., Heukamp L.C. (2008). Insulin-like growth factor-1 receptor acts as a growth regulator in synovial sarcoma. J. Pathol..

[168] de Bruijn D.R., Allander S.V., van Dijk A.H., Willemse M.P., Thijssen J., van Groningen J.J., Meltzer P.S., van Kessel A.G. (2006). The synovial-sarcoma-associated *SS18-SSX2* fusion protein induces epigenetic gene (de)regulation. Cancer Res..

[169] Olmos D., Postel-Vinay S., Molife L.R., Okuno S.H., Schuetze S.M., Paccagnella M.L., Batzel G.N., Yin D., Pritchard-Jones K., Judson I. (2010). Safety, pharmacokinetics, and preliminary activity of the anti-IGF-1R antibody figitumumab (CP-751,871) in patients with sarcoma and Ewing’s sarcoma: A phase 1 expansion cohort study. Lancet Oncol..

[170] Schöffski P., Adkins D., Blay J.Y., Gil T., Elias A.D., Rutkowski P., Pennock G.K., Youssoufian H., Gelderblom H., Willey R. (2013). An open-label, phase 2 study evaluating the efficacy and safety of the anti-IGF-1R antibody cixutumumab in patients with previously treated advanced or metastatic soft-tissue sarcoma or Ewing family of tumours. Eur. J. Cancer.

[171] Krieg A.H., Hefti F., Speth B.M., Jundt G., Guillou L., Exner U.G., von Hochstetter A.R., Cserhati M.D., Fuchs B., Mouhsine E. (2011). Synovial sarcomas usually metastasize after >5 years: A multicenter retrospective analysis with minimum follow-up of 10 years for survivors. Ann. Oncol..

[172] ten Heuvel S.E., Hoekstra H.J., Bastiaannet E., Suurmeijer A.J. (2009). The classic prognostic factors tumor stage, tumor size, and tumor grade are the strongest predictors of outcome in synovial sarcoma: No role for SSX fusion type or ezrin expression. Appl. Immunohistochem. Mol. Morphol..

[173] Machen S.K., Easley K.A., Goldblum J.R. (1999). Synovial sarcoma of the extremities: A clinicopathologic study of 34 cases, including semi-quantitative analysis of spindled, epithelial, and poorly differentiated areas. Am. J. Surg. Pathol..

[174] Stanelle E.J., Christison-Lagay E.R., Healey J.H., Singer S., Meyers P.A., La Quaglia M.P. (2013). Pediatric and adolescent synovial sarcoma: Multivariate analysis of prognostic factors and survival outcomes. Ann. Surg. Oncol..

[175] van der Graaf W.T.A., Orbach D., Judson I.R., Ferrari A. (2017). Soft tissue sarcomas in adolescents and young adults: A comparison with their paediatric and adult counterparts. Lancet Oncol..

[176] Kubo T., Shimose S., Fujimori J., Furuta T., Ochi M. (2015). Prognostic value of *SS18-SSX* fusion type in synovial sarcoma; systematic review and meta-analysis. SpringerPlus.

[177] Canter R.J., Qin L.X., Maki R.G., Brennan M.F., Ladanyi M., Singer S. (2008). A synovial sarcoma-specific preoperative nomogram supports a survival benefit to ifosfamide-based chemotherapy and improves risk stratification for patients. Clin. Cancer Res..

[178] Essary L.R., Vargas S.O., Fletcher C.D. (2002). Primary pleuropulmonary synovial sarcoma: Reappraisal of a recently described anatomic subset. Cancer.

[179] Kim G.H., Kim M.Y., Koo H.J., Song J.S., Choi C.M. (2015). Primary pulmonary synovial sarcoma in a tertiary referral center: Clinical characteristics, CT, and 18F-FDG PET findings, with pathologic correlations. Medicine.

[180] Hill L.A., Scott R.W., Martin L.A., Arostegui M., Davenport G., Vemon M., Hofvander J., Wang X.Q., Li J., Nielsen T.O. (2025). The fibroblast epigenome underlies SS18::SSX-mediated transformation in synovial sarcoma. Nat. Commun..

[181] Wang A.X., Jones K.B., Nielsen T.O. (2025). Molecular and epigenetic oncogenesis in synovial sarcoma: Implications for cancer biology, diagnosis and treatment. Oncogene.

